# Liver sinusoidal endothelial cells regulate the balance between hepatic immunosuppression and immunosurveillance

**DOI:** 10.3389/fimmu.2024.1497788

**Published:** 2025-01-17

**Authors:** Kimberly N. Kremer, Hadeel A. Khammash, Anjelica M. Miranda, Lauren N. Rutt, Shannon M. Twardy, Paige E. Anton, Margaret L. Campbell, Christian Garza-Ortiz, David J. Orlicky, Roberta Pelanda, Rebecca L. McCullough, Raul M. Torres

**Affiliations:** ^1^ Department of Immunology and Microbiology, University of Colorado School of Medicine, Aurora, CO, United States; ^2^ Department of Pharmaceutical Sciences, Skaggs School of Pharmacy and Pharmaceutical Sciences, Aurora, CO, United States; ^3^ Department of Pathology, University of Colorado School of Medicine, Aurora, CO, United States

**Keywords:** liver sinusoidal endothelial cells, T cell exhaustion, hepatocellular carcinoma, T cell, Immunosuppression

## Abstract

As a metabolic center, the liver prevents inappropriate immune responses to abundant dietary antigens within the liver that could result in liver injury. This self-preservation mechanism can however decrease the efficiency of immunosurveillance of malignant cells by CD8 T cells. Hepatocellular carcinoma (HCC) is initiated by chronic viral infections, chronic alcohol consumption, and/or a fatty diet that leads to liver injury, fibrosis, and cirrhosis. HCC patients have high levels of dysfunctional and exhausted T cells, however, it is unclear which stage of HCC development contributes to T cell dysfunction. Repair of liver injury is initiated by interactions between injured hepatocytes and liver sinusoidal endothelial cells (LSEC), however, chronic injury can lead to fibrosis. Here, using a diethylnitrosamine/carbon tetrachloride (DEN/CCl_4_) mouse model of early HCC development, we demonstrate that chronic liver injury and fibrosis are sufficient to induce a CD8 T cell exhaustion signature with a corresponding increase in expression of immunosuppressive molecules on LSEC. We show that LSEC alter T cell function at various stages of T cell differentiation/activation. LSEC compete with dendritic cells presenting the same antigen to naïve CD8 T cells resulting in a unique T cell phenotype. Furthermore, LSEC abrogate killing of target cells, in an antigen-dependent manner, by previously activated effector CD8 T cells, and LSEC change the effector cell cytokine profile. Moreover, LSEC induce functional T cell exhaustion under low dose chronic stimulation conditions. Thus, LSEC critically regulate the balance between preventing/limiting liver injury and permitting sufficient tumor immunosurveillance with normal hepatic functions likely contributing to HCC development under conditions of chronic liver insult.

## Introduction

Hepatic CD8 T cells isolated from patients with hepatocellular carcinoma (HCC) often display an exhausted signature that correlates with poor clinical outcomes. These exhausted hepatic CD8 T cells express high levels of programmed cell death protein 1 (PD-1) and inhibitory molecules such as T cell immunoglobulin and mucin-domain containing-3 (Tim3), T cell immunoglobulin and ITIM domain (TIGIT), cytotoxic T-lymphocyte-associated protein 4 (CTLA-4), and lymphocyte activation gene 3 (Lag3) (1–4). Moreover, the exhaustion signature most often correlates with decreased cytokine production and impaired cytolytic activity (2, 5). While HCC is the terminal stage of liver disease, HCC arises from multiple etiologies including hepatitis B/C, chronic alcohol consumption, or a fatty diet, which can lead to steatosis, fibrosis and cirrhosis (6, 7). However, it remains unclear when and how T cell exhaustion occurs during the progressive stages of liver disease. Here, we demonstrate that together liver injury and fibrosis are sufficient to induce T cell exhaustion and that ex vivo LSEC can induce both T cell dysfunction and T cell exhaustion. Together, these data suggest that LSEC act as critical gatekeepers to modulate T cell immunity in the liver microenvironment.

CD8 T cells activated by specific antigen presented by dendritic cells (DC) within draining lymph nodes upregulate CD44, PD-1, CD25, begin proliferating, and differentiate into cytotoxic effector cells that leave the lymph node in search of infected or malignant cells that present the cognate antigen. Upon finding target cells presenting cognate antigen, effector CD8 T cells secrete cytokines and/or kill the infected/malignant cell via a perforin-dependent mechanism, thus eliminating the infection or cancer (8, 9). However, viruses that induce chronic infections as well as many malignant cells have adapted mechanisms to suppress CD8 T cell effector functions to avoid elimination. These mechanisms include, but are not limited to, increased inhibitory molecule expression by the target cells, increased regulatory T cell (Treg) frequency, secretion of inhibitory cytokines (interleukin-10, IL-10, and transforming growth factor-β, TGFβ), and induction of T cell exhaustion by chronic stimulation with low dose antigen (4, 10–13). Much investigative effort has focused on characterizing these T cell inhibitory mechanisms for the purpose of developing immunotherapies that restore function to these dysfunctional T cells in order to eliminate malignant cells (8, 12, 14–17).

To be an effective metabolic center, the liver must also provide an immunologically tolerant environment to prevent an immune response to dietary antigens (18). The unique architecture of the liver, in particular the liver sinusoids, provide abundant opportunity for CD8 T cells to interact with a variety of antigen-presenting hepatic cells including hepatic stellate cells, Kupffer cells (KC), infiltrating macrophages (IM), and liver sinusoidal endothelial cells (LSEC) (18). The most abundant non-parenchymal cell (NPC) type, LSEC, line the sinusoids through which blood, and importantly CD8 T cells, flows at a low rate, providing ample opportunity for LSEC-T cell interactions (19–22). LSEC rely on scavenger and mannose receptors to capture and present circulating antigen at a 100-fold higher rate than DC *in vivo* (20). Via a PD-L1-dependent mechanism, and in the absence of CD80/86 costimulation, LSEC-mediated activation of CD8 T cells leads to T cell proliferation but impaired CD8 T cell cytokine production and killing, compared to CD8 T cells activated by DC (23, 24). Extremely high concentrations of antigen can override LSEC-mediated inhibition of CD8 T cell cytokine production, but not cytolytic activity unless exogenous IL-2 is provided (25). Additionally, DC-mediated activation of CD8 T cells is impaired by LSEC in an antigen-independent manner by an unknown mechanism (26). Interestingly, the soluble colorectal cancer antigen, carcinoembryonic antigen (CEA), injected into mice was preferentially taken up and presented by LSEC but not by DC; moreover, LSEC tolerized antigen-specific CD8 T cells to CEA which failed to control tumor growth (27). Thus, LSEC are critical regulators of T cell immunity within the liver.

Fibrosis occurs as a result of failure to repair liver injury, and is associated with a majority of HCC cases, with 70-90% of tumors developing in cirrhotic livers (6, 28, 29). While the early stages of fibrosis are reversible, chronic liver injury due to hepatitis B/C infections, chronic alcohol consumption, and/or a high fat diet can result in advanced fibrosis and cirrhosis (6, 14, 22). Although fibrosis induces capillarization of LSEC, which likely prevents interactions between CD8 T cells and the infected/malignant hepatocytes (30), the mechanisms that drive HCC progression from fibrosis development are unclear (29). Various mouse models exist to promote HCC with the intent to study different stages of HCC development and/or the outcomes (14, 31–33). Carbon tetrachloride (CCl_4_) is a widely used inducer of reproducible and predictable fibrosis, but, alone, does not induce HCC (14). Diethylnitrosamine (DEN) is a carcinogen that induces sufficient DNA damage to drive HCC development. However, DEN challenge alone can take nearly a year to lead to HCC and importantly, does not induce liver fibrosis, and, therefore, does not recapitulate human HCC development (14, 31, 34). However, the combination of DEN with CCl_4_ treatment (DEN/CCl_4_ model) provides a reproducible model of HCC that evolves from liver injury and fibrosis (32, 35–40), with fibrosis being the most common factor among HCC developing from different etiologies (6, 28, 29). Moreover, this DEN/CCl_4_ model uncouples the effects of chronic infection, steatosis, and alcohol-induced damage from fibrosis in order to allow a more clear characterization of how fibrosis contributes to HCC development.

In this report, we show, using the DEN/CCl_4_ model, that liver injury and fibrosis coincides with the phenotypic exhaustion of CD8 and CD4 T cells as well as a corresponding increase in expression of immunosuppressive molecules by LSEC. Moreover, we demonstrate that LSEC induce CD8 T cell dysfunction at varying stages of T cell differentiation/activation. Our results show that LSEC impair DC-mediated activation of CD8 T cells, abrogate the ability of previously activated CD8 effector T cells to kill target cells, and induce exhaustion of T cells. Thus, LSEC, the most abundant NPC in the liver, play a key role in regulating T cell-mediated immunosurveillance. Furthermore, the immunosuppressive abilities of LSEC are enhanced by fibrosis and likely result in increased T cell dysfunction and subsequent HCC development.

## Materials and methods

### Mice

C57BL/6J (stock no. 000664, The Jackson Laboratory) and OT-I mice (stock no.003831) were bred and housed at the University of Colorado Anschutz Medical Campus vivarium (Aurora, CO). All procedures with animals were approved by the University of Colorado Institutional Animal Care and Use Committee.

### DEN/CCl_4_ model and histopathological analysis

Male mice were used for this study because males (both human and mouse) are more prone to HCC development than females (32, 35, 38, 39). At 14 days old, male C57BL/6J mice were injected IP with 25mg/kg diethylnitrosamine (DEN, Sigma, 73861), a genotoxic agent. At 8 weeks of age, mice received twice weekly i.p. injections of 0.25μl/g of carbon tetrachloride (CCl_4_, Sigma, 270652) or the vehicle (olive oil, Sigma, O1514) for 8 weeks. At ~16 weeks of age, mice were anesthetized, blood was taken from the inferior vena cava, and the livers were harvested and divided for analysis with the right median lobe utilized for IHC and the majority of the remainder of the liver used for immunophenotypic analysis by flow cytometry. For histological analysis, formalin-fixed livers were sectioned at 4 microns, embedded in paraffin and stained with hematoxylin and eosin or with Picrosirius Red. Scoring of liver pathology used procedures adapted for mice as previously described (41, 42) similar to the validated human liver histological scoring system established by Kleiner et al. (43) Briefly, hepatocyte death (hepatocyte ballooning, acidophilic bodies, and necrotic cells), the presence of various inflammatory cells and foci, reactive tissue changes (e.g., ductal reaction, glycogenated nuclei, increased numbers of mitotic figures), and the extent of steatosis were determined and scored by a trained histopathologist that was blinded to the treatments and grouping of the mice. Histologic images were captured on an Olympus BX51 microscope equipped with a DP73 digital camera (Olympus) using the Cell Sense Application Program. All images were cropped and assembled using Photoshop CS2 (Adobe Systems, Inc.; Mountain View, CA). To quantify the fibrillar collagen content, PicroSirius Red staining was performed. Approximately five 40x polarized light images were “tiled” across the stained tissue, then the images were imported into SlideBook (Intelligent Imaging Innovations, Denver, Colorado), and the positive pixels were quantified. Data is expressed as the percentage of pixels that are positive. Sections were then assessed for hyperplasia, adenocarcinoma and HCC lesions as previously described (44, 45) and in close association with the scoring found on the National Toxicology Program website.

### Immunophenotypic analysis

The livers from mice treated with olive oil/CCl_4_ or DEN/CCl_4_ were digested as in Finlon et al. (46) Briefly, the livers were chopped and digested in Click’s media (Sigma) with a final concentration of 0.25mg/ml DNAse and 625u/ml Collagenase IV for 30 min. The digested liver was placed through a 100μm strainer and rinsed with liver isolation buffer (4.8% BSA and 2mM EDTA in HBSS) followed by erythrocyte lysis. Nonparenchymal cells were then isolated with a 20% Optiprep (Sigma) gradient, rinsed and flow cytometrically analyzed. Cells were stained with anti-mouse antibodies (clones in parentheses) from Biolegend, unless otherwise indicated, for CD45 (30-F11), CD3 (17A2), CD8 (53-6.7), CD4(GK1.5), FoxP3 (MF-14), CD146 (ME-9F1), CD31 (390), PDPN (8.1.1), CD14 (Sa14-2), F4/80 (BM8), CD11b (M1/70), PD-L1 (10F.9G2), ICAM-1 (R&D, 166623), H2-K^b^ (AF6-88.5), I-A^b^ (AF6-120.1), FasL (MFL3), CD80 (16-10A1), CD44 (IM7), PD-1 (eBioscience, J43), TIGIT (1G9), Tim3 (RMT3-23), CD25 (eBioscience, PC61.5). FoxP3 was analyzed using the eBioscience FoxP3 staining set according to manufacturer’s instructions.

### *In vitro* activation of naïve CD8 T cells

Naïve C57BL/6J mice were anesthetized and livers were perfused with PBS. The livers were digested and NPC were isolated as described above. LSEC were isolated using Miltenyi microbeads by CD45 negative selection (130-052-301) followed by CD146 positive selection (130-092-007), with a purity of >95%. LSEC were plated at 0.2 x 10 (6) cells per well on collagen-coated plates in complete media (RPMI, 10% FBS, L-glutamine, penicillin-streptomycin, non-essential amino acids, sodium pyruvate, and b-mercaptoethanol) for 24 hr prior to the start of cocultures. For DC, spleens of naïve C57BL/6J mice were harvested, digested in Click’s media with 0.25 μg/ml DNAse and 100u/ml Collagenase IV for 20 min, and placed through a 100μm strainer followed by erythrocyte lysis. DC were then isolated via CD11c positive selection (Miltenyi, 130-125-835). Naïve OT-I T cells were isolated from erythrocyte-lysed spleens via the CD8 T cell isolation kit II (Miltenyi, 130-104-075) and stained with CellTrace Violet (Invitrogen, C34557) for analysis of proliferation. For cocultures, LSEC received fresh complete media, and then 200,000 splenic DC and 500,000 naïve OT-I CD8 T cells were added with 2μg/ml SIINFEKL (N4), and the cells were cultured for 72 hr prior to harvest of cells for flow analysis and supernatants for analysis of IL-2 (555148), IFNγ (551866), and TNF (558534) by BD Biosciences ELISAs.

### Effector CD8 T cell killing assay

OT-I CD8 effector T cells were generated as previously described (47). Briefly, OT-I CD8 T cells were stimulated in an antigen-specific manner by treating erythrocyte-lysed splenocytes with 2 μg/mL N4 peptide in complete media for 3 days. OT-I CD8 T cells were then cultured in fresh media with IL-2 at 100 u/mL for an additional 3 days. Peptide-activated CD8+ T cells were isolated via Ficoll separation. For target cells, erythrocyte-lysed splenocytes were labeled as either CFSE-hi or CFSE-lo. CFSE-hi cells were pulsed with 2μg/ml N4 peptide for 1 hr, washed and then combined with unpulsed CFSE-lo cells. Prior to coculture set up, OT-I effector cells were serially diluted and then combined with 2 x 10^5^ N4- or GP33-pulsed LSEC (isolated and cultured 24 hr earlier) for 30 min at 37C. Then 4 x 10^5^ CFSE-labeled targets were added, and the cells were cultured for 6 hr prior to harvest of cells for flow cytometry and the supernatants for IFNγ analysis as above. Killing of target cells was assessed by gating on live, single, CFSE^+^ cells and calculating the ratio of CFSE^hi^ (N4-pulsed targets) to CFSE^lo^ (unpulsed targets) cells.

### *In vitro* model of chronic stimulation

The chronic stimulation assay was based on Wu et al. (48) LSEC, splenic DC, and naïve OT-I T cells were isolated as described above. LSEC (isolated and plated 24 hr earlier) and freshly isolated DC were separately treated with 200nM N4 for 30 minutes, and then washed prior to the addition of 2 x 10^5^ of naïve OT-I CD8 T cells. On Day 2, cells were replated at 2 x 10^5^ per well with 10u/ml IL-2 and either 2nM N4 (low dose chronic stimulation) or no peptide (acute stimulation). On Days 6 and 8, cells were replated at 4 x 10^5^ per well with IL-2 and either N4 or no peptide as on Day 2. On Day 9, cells were either harvested for phenotypic T cell exhaustion via flow cytometry assaying CD44, PD-1, TIGIT, and Tim3 or restimulated for functional T cell exhaustion analysis assayed by intracellular cytokine staining. Restimulation of both chronically and acutely stimulated T cells consisted of the addition of 800nM N4, 100u/ml IL-2, Golgistop, and Golgiplug for 5 hr prior to harvest for intracellular cytokine staining using eBiosciences Fixation and Permeabilization buffer set (88-8824-00) and IL-2 (clone JES6-5H4), IFNγ (clone XMG1.2), and TNF (clone MP6-XT22) from Biolegend.

### Statistical analysis

Results are displayed as means ± SEM. P value was considered significant if P ≤ 0.05. To determine significance, ratios and percentages were transformed to log form prior to statistical analysis. Data that were normally distributed were analyzed with a paired or unpaired t test, while a Welch’s correction was added to data sets that displayed unequal variance. Data sets that were not normally distributed were analyzed with the non-parametric Mann-Whitney test. Paired t tests were used for most *in vitro* experiments, while unpaired t tests were used for cellular analysis from mouse harvests. The one-sample t test was used for *in vitro* experiments with high technical variation that were normalized to a control cell response between experimental replicates, with a theoretical value of 1. Independent experiments were executed for each dataset as indicated in the figure legends. The statistical analysis used for each experiment is indicated in the figure legends. Analysis was performed using GraphPad Prism version 9.0 and/or Excel.

## Results

### The DEN/CCl_4_ mouse model induces liver injury, fibrosis, and early stages of HCC development

**Figure 1:**
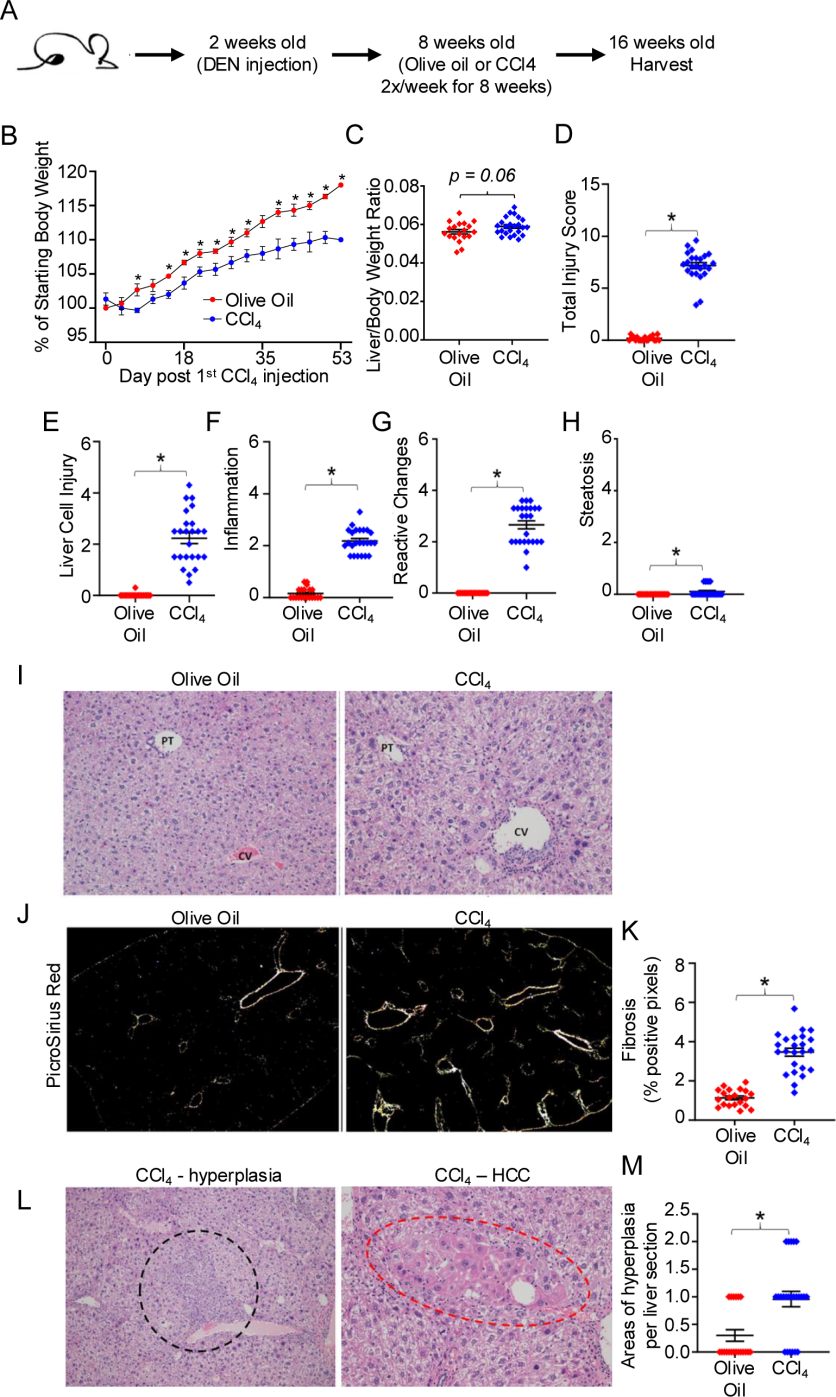
The DEN/CCl_4_ mouse model induces liver injury, fibrosis, and early stages of HCC development. **(A)** C57BL/6 mice were injected at 14 days old with 25mg/kg DEN, and then beginning at 8 weeks of age, received twice weekly IP injections of 0.25μl/g CCl_4_ or of olive oil (control) for 8 weeks. **(B)** Mouse weights measured every 3-4 days for DEN/olive oil treated (red) or DEN/CCl_4_-treated (blue). Mice were sacrificed at ~16 weeks of age, and **(C)** the livers weighed, and then formalin-fixed for further histopathological analysis of **(D)** total liver injury, **(E)** liver cell injury, **(F)** inflammation, **(G)** reactive changes, and **(H)** steatosis as described in Materials and Methods. Representative images of **(I)** H&E staining (central vein (CV) and portal triad (PT), **(J)** Picrosirius Red staining, and **(L)** areas of hyperplasia/HCC, respectively. Summary graphs of **(K)** fibrosis and **(M)** areas of hyperplasia. Each dot denotes a mouse, ± SEM. *P ≤ 0.05, Student’s unpaired t test for B, C and Mann-Whitney test for D-H, K, M. n = 3 independent experiments, with 6-8 mice per experiment.

HCC has been characterized to harbor exhausted T cells and an increased frequency of Tregs (1–3, 6, 10–12), however, very little is known about which stage of HCC development induces T cell exhaustion and increases Treg frequency. We sought to interrogate the early stages of HCC development, after liver injury and fibrosis have been initiated and malignant cells are just starting to emerge by histological analysis but are not yet macroscopically visible. Thus, we utilized a low dose DEN/CCl_4_ mouse model in C57BL/6J mice that uncouples fibrosis from other T cell exhaustion inducing factors including hepatitis B/C infection and a high fat diet (32, 38, 39). The DEN/CCl_4_ model, which has been used in diverse mouse strains and at various doses, can lead to 100% of male mice developing tumors over 14 weeks of CCl_4_ treatment (32, 35, 36, 38–40). Thus, in our study C57BL/6J mice were harvested after just 8 weeks of CCl_4_ treatment in order to focus on the role of liver injury and fibrosis on the hepatic immunophenotype prior to tumor development. Male mice were used for these experiments, as males (human and mouse) have a higher propensity to develop HCC than females (32, 35, 38, 39). Male mice were injected at 2 weeks of age with a single dose of 25 mg/kg DEN. At 8 weeks of age, mice were subsequently injected twice weekly with 0.25 μl/g CCl_4_ or olive oil (control) for 8 weeks (**Figure 1A**). As shown in **Figure 1B**, mice treated with DEN/CCl_4_ gained significantly less weight than the control mice treated with olive oil as vehicle control. Mice treated with DEN/CCl_4_ had increased, but not significantly, liver to body weight ratio (**Figure 1C**). Nonetheless, DEN/CCl_4_-treated mice did display significantly more liver injury compared to DEN/olive oil-treated mice, as indicated by blind histological analysis of liver cell injury, inflammation, reactive changes and steatosis (**Figures 1D–I**). Mice treated with DEN/CCl_4_ also displayed significantly increased fibrosis as measured by Picrosirius red compared to DEN/Olive oil-treated mice (**Figures 1J, K**). Although areas of hyperplasia are not always indicative of cancer, DEN/CCl_4_ mice showed a significant increase in the number of hyperplastic regions per liver section compared to control mice (**Figures 1L, M**). Finally, areas of adenoma/HCC (**Figure 1L**) were histologically identified in 3 of the 44 mice included in this study, indicating, that at this timepoint, early HCC is developing but was not yet detectable in a majority of the mice. These results indicate that the DEN/CCl_4_ model used here results in liver injury and fibrosis that precedes the development of HCC, and therefore is a good model to assess the role of liver injury and fibrosis on the hepatic immunophenotype prior to tumor development.

### The DEN/CCl_4_ mouse model induces an exhausted phenotype in hepatic CD8 and CD4 T cells as well as an increase in the frequency of Tregs in the liver

**Figure 2:**
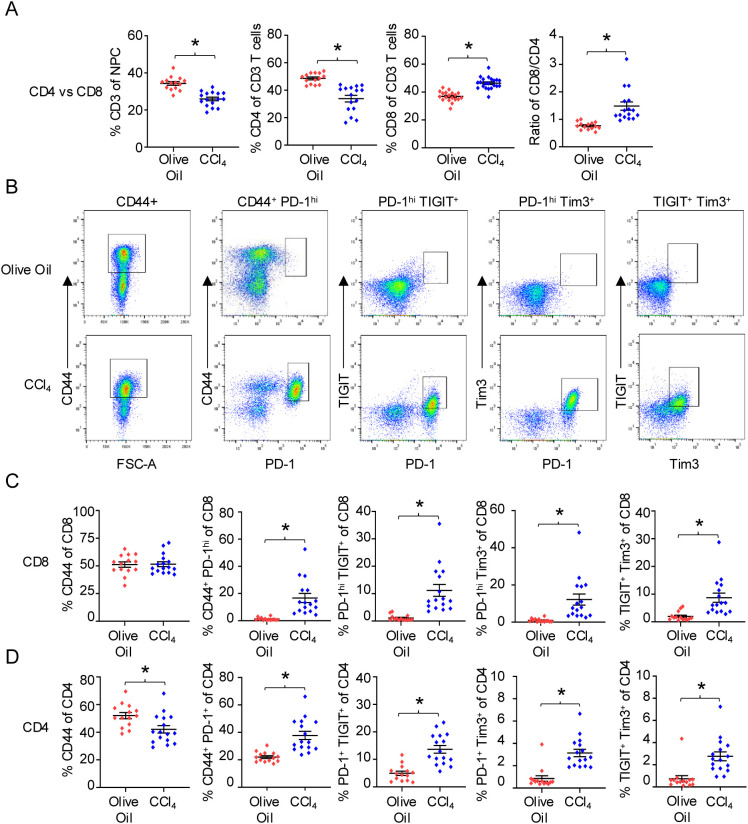
*The DEN/CCl_4_ mouse model induces an exhausted phenotype in hepatic CD8 and CD4 T cells.* On completion of DEN/CCl_4_ treatment, livers were harvested and digested, and NPC isolated and analyzed via flow cytometry. **(A)** Frequencies of hepatic CD3, CD4, and CD8 T cells in DEN/olive oil- (red) and DEN/CCl_4_-treated (blue) mice. **(B)** Representative flow cytometric plots of NPC gated on CD8^+^ T cells and analyzed for CD44, PD-1, TIGIT, and/or Tim3 frequencies. Panels **(C, D)** provide summary frequencies of CD44^+^, CD44^+^PD-1^hi^, PD-1^hi^TIGIT^+^, PD-1^hi^ Tim3^+^, and TIGIT^+^Tim3^+^ for **(C)** CD8 and **(D)** CD4 T cells between DEN/olive oil- (red) and DEN/CCl_4_-treated (blue) mice with each dot denoting a mouse, ± SEM. *P ≤ 0.05, Student’s unpaired t test or Mann-Whitney test, n = 2 independent experiments, with 6-8 mice per experiment.

To understand the hepatic T cell phenotype within an injured and fibrotic liver, we harvested and digested the livers from both DEN/olive oil- and DEN/CCl_4_-treated mice, isolated the NPC, and analyzed the hepatic T cell phenotype via flow cytometry. DEN/CCl_4_ treatment resulted in a significant decrease in the percent of CD3^+^ T cells among NPC in the liver, compared to DEN/olive oil-treated mice (**Figure 2A**). Among the hepatic T cells from DEN/CCl_4_-treated mice, there was a significant decrease in the percent of CD4 T cells present with a corresponding increase in the percent of CD8 T cells, thus resulting in a significantly higher CD8/CD4 ratio (**Figure 2A**). Of the CD8 T cells, there was no change in the (~50%) percent of activated CD44^+^ cells present between the two groups, however, DEN/CCl_4_ treatment induced a significant increase in the proportion of CD44^+^PD-1^hi^ CD8 T cells compared to control mice (**Figures 2B, C**). Additionally, we evaluated the expression of the well-characterized exhaustion markers TIGIT and Tim3 to assess hepatic T cell exhaustion (1–4), where TIGIT has been shown to be more commonly expressed in exhausted T cells that reside within the liver compared to Tim3, a universal exhaustion marker (15–17, 49). Importantly, the percentage of PD-1^hi^TIGIT^+^, PD-1^hi^Tim3^+^ and TIGIT^+^Tim3^+^ CD8 T cell populations were all significantly increased in DEN/CCl_4_ treated livers compared to olive oil-treated livers (**Figures 2B, C**). Thus, DEN/CCl_4_ treatment results in a higher percentage of hepatic CD8 T cells in the liver with an exhausted phenotype compared to control mice. In contrast to CD8 T cells, there was a lower percentage of CD4 T cells expressing CD44 in DEN/CCl_4_-treated mice compared to control mice (**Figure 2D**). Nonetheless, DEN/CCl_4_-treated mice displayed a similar significant increase in the percent of CD44^+^PD-1^+^, PD-1^+^TIGIT^+^, PD-1^+^Tim3^+^, and TIGIT^+^Tim3^+^ CD4 T cell populations compared to the olive oil-treated control mice (**Figure 2D**), indicating that DEN/CCl_4_ treatment also promotes a CD4 T cell exhaustion phenotype. Notably, DEN/CCl_4_ treatment led to an almost doubling of the percent of CD4^+^FoxP3^+^ T cells in the liver compared to control mice (15% vs 7%; **Supplementary Figure 1**); this population potentially represents Tregs, though further analysis is required. Together, results presented in **Figure 1** and **Figure 2** show that DEN/CCl_4_ treatment increases not only the percent of phenotypically exhausted CD8 and CD4 T cells but also results in an increased presence of CD4^+^FoxP3^+^ T cells. Thus, fibrosis coincides with an immunosuppressed environment, despite the lack of histological evidence of HCC development in the vast majority (93%; 41 of 44 mice) of the (control and DEN/CCl_4_-treated) mice.

### Expression of immunosuppressive molecules are increased on LSEC by DEN/CCl_4_ treatment

**Figure 3:**
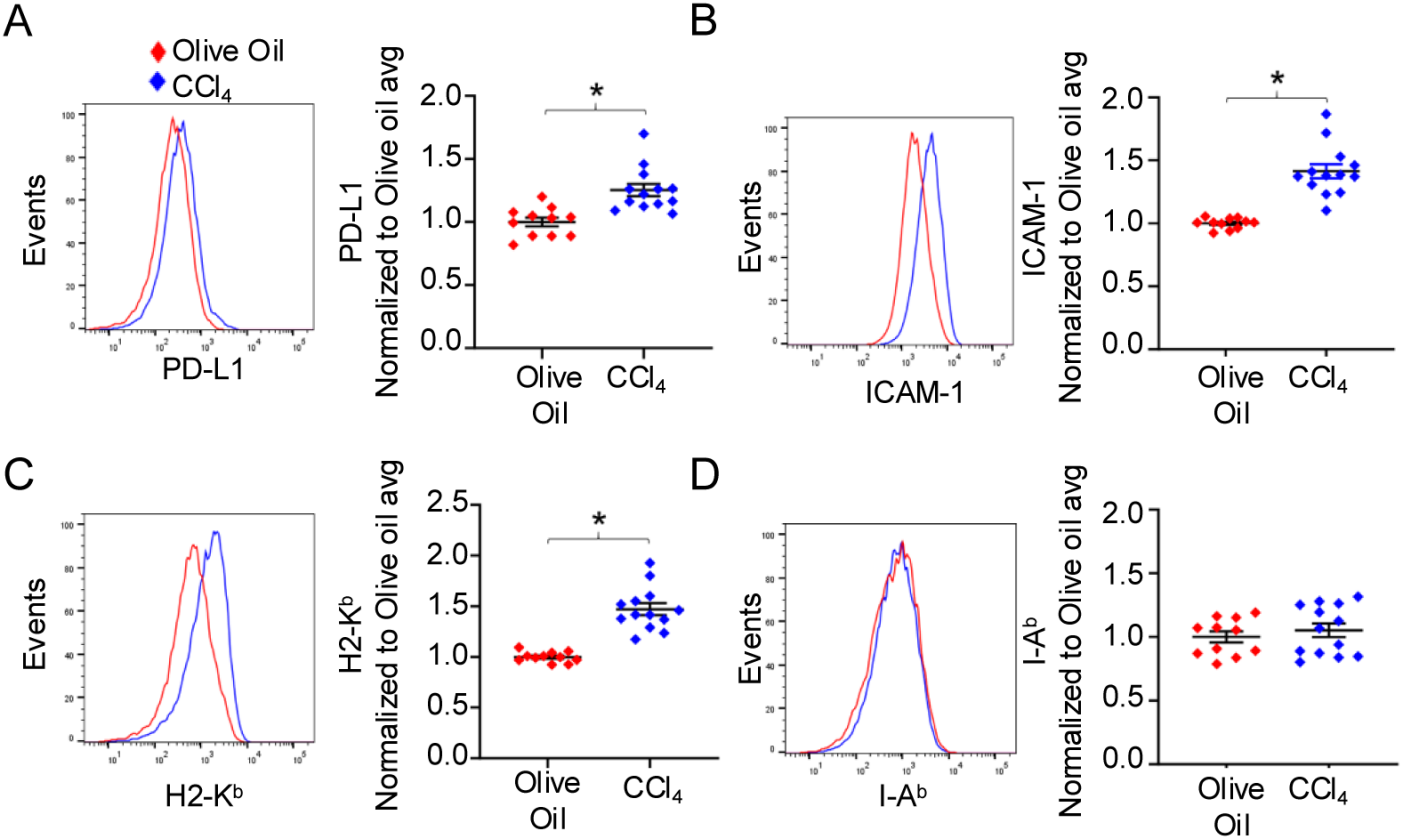
*Expression of immunosuppressive molecules are increased on LSEC by DEN/CCl_4_ treatment.* NPC from DEN/olive oil- (red) or DEN/CCl_4_-treated (blue) mice were isolated as in **Figure 2** and the expression of immunosuppressive molecules by LSEC (gated as CD45^-^CD146^+^CD31^+^PDPN^-^) determined by flow cytometric analysis. A representative histogram plot and a summary graph are shown for **(A)** PD-L1, **(B)** ICAM-1, **(C)** H2-K^b^ and **(D)** I-A^b^ expression on LSEC. Each dot denotes a mouse, values are normalized to the DEN/Olive oil average, ± SEM. *P ≤ 0.05, Student’s unpaired t test, n = 2 independent experiments, with 5-8 mice per experiment.

Various cell types in the liver can act to suppress T cell function including LSEC, KC, and infiltrating monocyte-derived macrophages (IM) via different mechanisms including PD-L1, ICAM-1, and altered MHC expression (23–25, 27, 50–57). To determine the intercellular mechanisms driving T cell dysfunction in the DEN/CCl_4_ model, we first assessed the expression of immune regulatory molecules on LSEC (gated as CD45^-^CD146^+^CD31^+^PDPN^-^) (26, 46) among NPC from the livers of olive oil- or DEN/CCl_4_-treated mice. Results from these analyses revealed a significant increase in PD-L1, ICAM-1, and H2-K^b^ (MHC class I molecule MHC-I) expression, but not I-A^b^ (MHC class II molecule, MHC-II) on LSEC from livers of DEN/CCl_4_-treated mice compared to control mice (**Figures 3A–D**). In contrast, KC (gated as CD45^+^CD14^+^CD11b^lo^F4/80^hi^) (58) from DEN/CCl_4_-treated mice displayed significantly decreased expression of PD-L1, ICAM-1, H2-K^b^ and I-A^b^, in addition to the activation marker CD80 compared to control mice (**Supplementary Figures 2A, B**). The expression of FasL, the TNF superfamily member that induces apoptosis of T cells expressing Fas (18), did not change on KC between groups. IM (gated as CD45^+^CD14^+^CD11b^hi^F4/80^lo^) (58) from DEN/CCl_4_-treated livers displayed a modest but significant increase in the immunosuppressive molecule, PD-L1, and the activation marker, CD80, however, ICAM-1, H2-K^b^, I-A^b^, and FasL expression did not change between treatment groups (**Supplementary Figure 2C**). Together, the significant increase in expression of immunosuppressive molecules on LSEC, but not KC or IM, suggests that LSEC mediate the fibrosis-induced exhaustion of hepatic CD8 and CD4 T cells upon DEN/CCl_4_ treatment to mice, prior to HCC development.

### LSEC limit DC-mediated T cell activation

**Figure 4:**
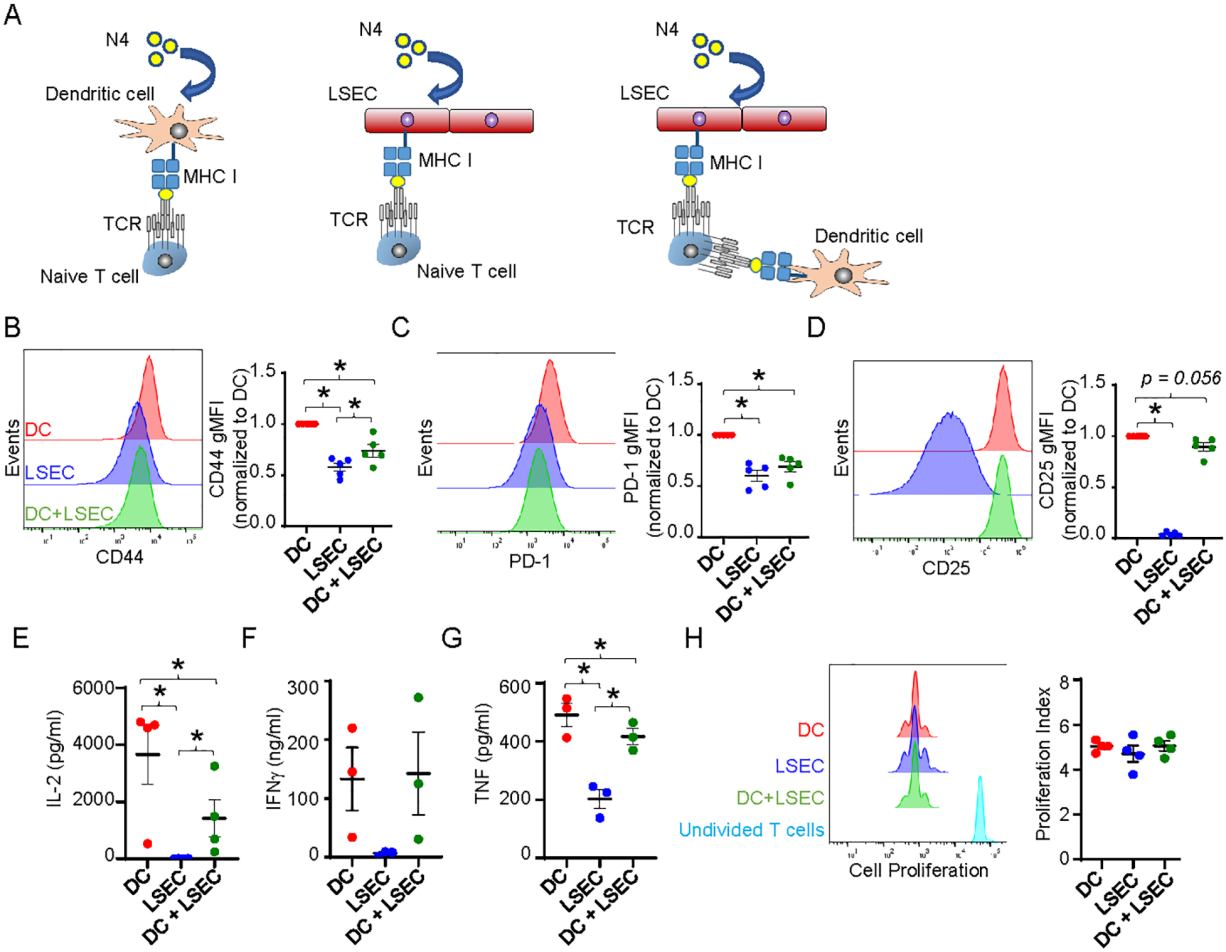
LSEC limit DC-mediated T cell activation. **(A)** Schematic of CTV-labeled naïve OT-I CD8 T cells cocultured in the presence of 2μg/ml N4 with primary splenic DC, primary LSEC, or splenic DC and LSEC. **(B–D)** After 72 hours of culture, the cells were harvested and analyzed via flow cytometry for **(B)** CD44, **(C)** PD-1, and **(D)** CD25 expression on OT-I CD8 T cells after culture with DC (red), LSEC (blue), or both DC and LSEC (green). A representative histogram (left) and a summary graph (right) are shown for each coculture condition, ± SEM. *P ≤ 0.05, Student’s paired t test or one sample t test (with theoretical value of 1), n = 5 independent experiments. E-G) After 72 hr of culture, supernatants were harvested and analyzed for the secretion of **(E)** IL-2, **(F)** IFNγ, and **(G)** TNF, ± SEM. *P ≤ 0.05, Student’s paired t test, n = 3-4. **(H)** After 72 hrs of culture, cells were harvested and analyzed for proliferation via CTV dilution using FlowJo software. A representative histogram and a summary graph displaying the proliferation index are shown, ± SEM. CTV staining for non-stimulated T cells is shown in turquoise. *P ≤ 0.05, Student’s paired t test, n = 3.

Previous studies have shown that although LSEC are able to cross-present antigen to T cells similar to DC, LSEC fail to activate T cells comparably to DC. While LSEC can induce proliferation of T cells, these T cells fail to effectively upregulate activation markers, produce cytokines, and/or lyse target cells as effectively as T cells activated by DC (23–25, 27, 50). Coculture of naïve T cells with LSEC, that are unable to present cognate antigen due to mismatch MHC, has further been shown to impede DC-mediated T cell activation by an unidentified cell contact mechanism (26). However, we reason that the close proximity of DC, primarily found around the portal triad (59, 60), and LSEC, which line the hepatic sinusoids (19), to each other within the liver suggest that DC and LSEC might compete for the uptake and presentation of liver antigens to naïve T cells. Accordingly, we sought to determine the extent of T cell activation when LSEC and DC are both presenting the same antigen and competing for T cell interactions. To assess this, we employed a coculture system that activates naïve T cells in an antigen-specific manner and consisted of isolated primary naïve CD8 T cells cultured with primary hepatic LSEC and/or splenic DC. CD8 OT-I T cells, expressing a TCR transgene receptor, were used to assess antigen-dependent activation by the chicken ovalbumin (OVA) peptide, SIINFEKL (referred to here as N4) (61). Isolated naïve OT-I T cells were combined into 3 different coculture systems with either splenic DC or primary LSEC alone, or splenic DC and primary LSEC combined, and in the presence of specific N4 peptide in all cultures (**Figure 4A**). LSEC and DC were combined in a 1:1 ratio heavily favoring DC-mediated T cell activation, given only 1% of the nonparenchymal cells are DC (60) while LSEC comprise 15-20% of all hepatic cells (19). After 72 hr, CD8 T cells cocultured with N4-pulsed DC or LSEC significantly increased the expression of the activation markers, CD44, PD-1, and CD25 (**Figures 4B–D**), as previously reported, but the degree of activation mediated by LSEC was lower relative to that mediated by DC. Importantly, and not previously assessed, CD8 T cells cocultured with N4-pulsed LSEC and DC together had significantly decreased levels of CD44 and PD-1, compared to CD8 T cells cultured with DC alone (**Figures 4B, C**), while CD25 expression was less affected by LSEC and DC coculture (**Figure 4D**). These results suggest that LSEC impair the ability of DC to fully activate and promote effector function of CD8 T cells effectively. To investigate this further, we also measured cytokine production from these different coculture supernatants after 72 hrs by ELISA. These results revealed significantly more IL-2, IFNγ, and TNF by T cell coculture with DC alone compared to T cell coculture with LSEC alone (**Figures 4E–G**), as previously reported (24, 25). However, we further found that significantly less IL-2 and TNF, but not IFNγ, were produced when CD8 T cells were cocultured with DC and LSEC together compared to DC alone (**Figures 4E–G**). This decrease in cytokine production was not due to fewer T cells as a result of a diminished proliferation of the CD8 T cells during the 72 hrs of coculture, as measured by cell trace violet (CTV), between the different cocultures (**Figure 4H**). Together these results indicate that when LSEC and DC are presenting the same antigen simultaneously, naïve CD8 T cell activation is compromised resulting in decreased IL-2 and TNF secretion, but unaltered cell proliferation or IFNγ release. Thus, LSEC selectively alter DC-induced T cell upregulation of certain activation markers and the T cell cytokine secretion profile.

### LSEC potently inhibit CD8 effector T cell cytotoxicity while substantially increasing IFNγ production

**Figure 5:**
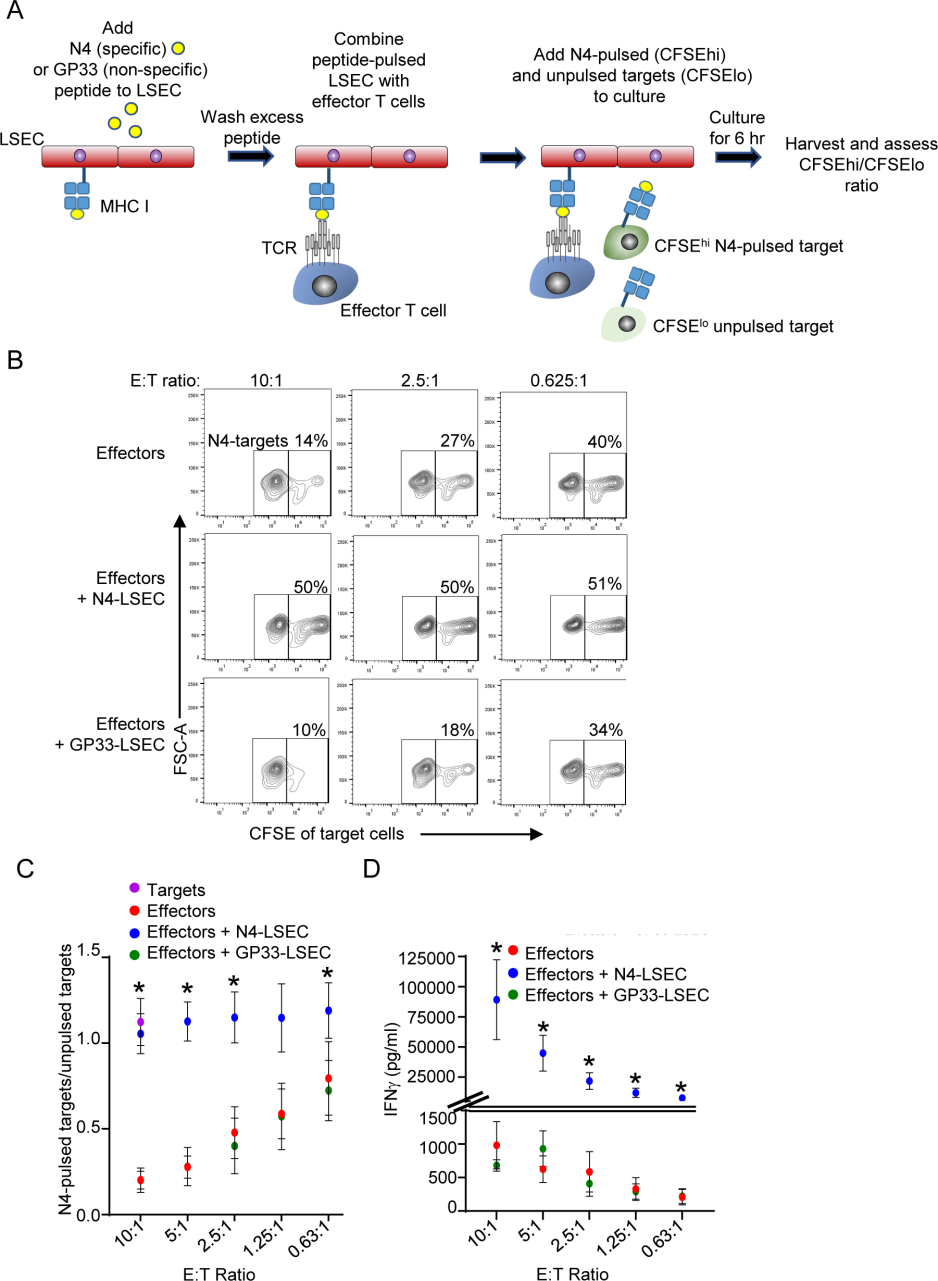
*LSEC potently inhibit CD8 effector T cell cytotoxicity while substantially increasing IFNγ production.* **(A)** Schematic of cytotoxic assay depicting LSEC pulsed with either N4 or GP33 (control) peptides and cocultured with serially diluted OT-I effector CD8 T cells for 30 minutes prior to addition of target cells presenting either N4-specific peptide (N4-pulsed) or not (un-pulsed). Target cells (C57BL/6J splenocytes) were differentially labeled as CFSE^hi^ or CFSE^lo^, and CFSE^hi^ cells were pulsed with N4, washed and combined with unpulsed CFSE^lo^ cells. After addition of the targets, the cocultures were maintained at 37C for 6 hr and then the cells were analyzed by flow cytometry. **(B)** Representative contour plots show the percent of N4-targets (CFSE^hi^) remaining of total CFSE+ cells at E:T ratios of 10:1 (left), 2.5:1 (middle), or 0.625:1 (right) for effector cells combined with target cells alone (top row), or together with N4-pulsed LSEC (middle row), or GP33-pulsed LSEC (bottom row). **(C)** Summary of OT-I CD8 T cell antigen-specific killing of target cells at decreasing E:T ratio shown as the ratio of N4-pulsed targets/unpulsed targets alone (purple dot) or in culture with effectors alone (red dots), effectors + N4-pulsed LSEC (blue dots), or effectors + GP33-pulsed LSEC (green dots), ± SEM. * denotes significant difference between targets cells cultured with effectors + N4-LSEC vs effectors alone or effectors + GP33-LSEC, P ≤ 0.05, Student’s paired t test, n = 4 independent experiments. **(D)** Culture supernatants were also analyzed for IFNγ production via ELISA after 6 hr of coculture and concentrations shown at different E:T ratios, ± SEM. * denotes significant difference between targets cells cultured with effectors + N4-LSEC (blue dots) vs effectors alone (red dots) or effectors + GP33-LSEC (green dots). Note: Overlap of some data points for effectors alone and effectors + GP33-LSEC obscure each other, however, all data points are contained in the graphs. P ≤ 0.05, Student’s paired t test, n = 3 independent experiments.

Naïve T cells are often activated in the draining lymph node by DC that have picked up antigen at the site of malignant cell development and subsequently migrate to the draining lymph node to present the antigen to naïve T cells (8, 9). We next asked if LSEC alter functions of effector cytotoxic CD8 T cells previously activated by lymphoid organ antigen presenting cells. To address this, we investigated whether LSEC alter the ability of effector cytotoxic T cells to kill target cells *in vitro*. Effector cytotoxic CD8 T cells were generated by culturing isolated naïve CD8 OT-I T cells with N4-pulsed splenocytes for 3 days, and then resting the stimulated cells in the presence of IL-2 for 3 additional days (47). To assess the cytolytic activity of the effector CD8 T cells in the absence of LSEC, we cocultured these *in vitro* generated effector cytotoxic CD8 T cells at a 1:1 ratio with splenocytes treated either with N4 peptide or left untreated and distinguished by either high or low, respectively, concentrations of CFSE labeling (**Figure 5A**). Furthermore, effector CD8 T cells were cocultured with decreasing effector:target cell (E:T) ratios. Effector CD8 T cells were cultured for 6 hours with targets cells, and effector cell cytolytic activity was subsequently assessed by flow cytometric analysis. The percent of N4-peptide pulsed (CFSE^hi^) targets vs unpulsed (CFSE^lo^) targets was determined after gating on all live CFSE-labeled target cells. **Figure 5B** shows that an E:T ratio of 10:1 results in target cell specific killing with only ~14% of N4-pulsed targets remaining from the 50% originally placed in culture. As expected, decreasing the E:T ratio increased the percent of N4-pulsed targets (**Figure 5B**) alive after coculture, as there are fewer effector cells per target. To assess the effect of LSEC specific antigen presentation on CD8 effector T cell cytolytic activity, we cultured CD8 T effector cells with N4-pulsed LSEC for 30 minutes prior to adding the target cells for 6 hr (**Figure 5A**). As shown in **Figures 5B, C**, LSEC presenting the N4 antigen to effector CD8 T cells potently impaired the killing ability of the effector T cells as indicated by a consistent 1:1 ratio of specific (N4-pulsed) to non-specific (unpulsed) target cells despite a high E:T ratio. Furthermore, the LSEC-mediated inhibition of CD8 T cell cytolytic activity was antigen-dependent, since LSEC pulsed with GP33, an irrelevant lymphocytic choriomeningitis virus (LCMV)-derived peptide (62), did not impair OT-I-specific effector cell killing. Interestingly, IFNγ production, assayed by ELISA from these same 6 hr cocultures, was significantly increased in the presence of LSEC presenting specific peptide, but not non-specific peptide, by ~50-100 fold in an E:T ratio dependent manner (**Figure 5D**). Together these results indicate that LSEC, presenting cognate antigen to effector cytotoxic CD8 T cells, potently impair CD8 T cell cytotoxicity, while simultaneously increasing IFNγ production by these same CD8 T cells. Considered together, these results suggest that LSEC presenting tumor antigens, in the vicinity of the malignant cells producing the same antigens, impede CD8 effector T cell ability to eliminate malignant cells within the liver, thus facilitating tumor progression.

### LSEC induce T cell exhaustion in CD8 T cells

**Figure 6:**
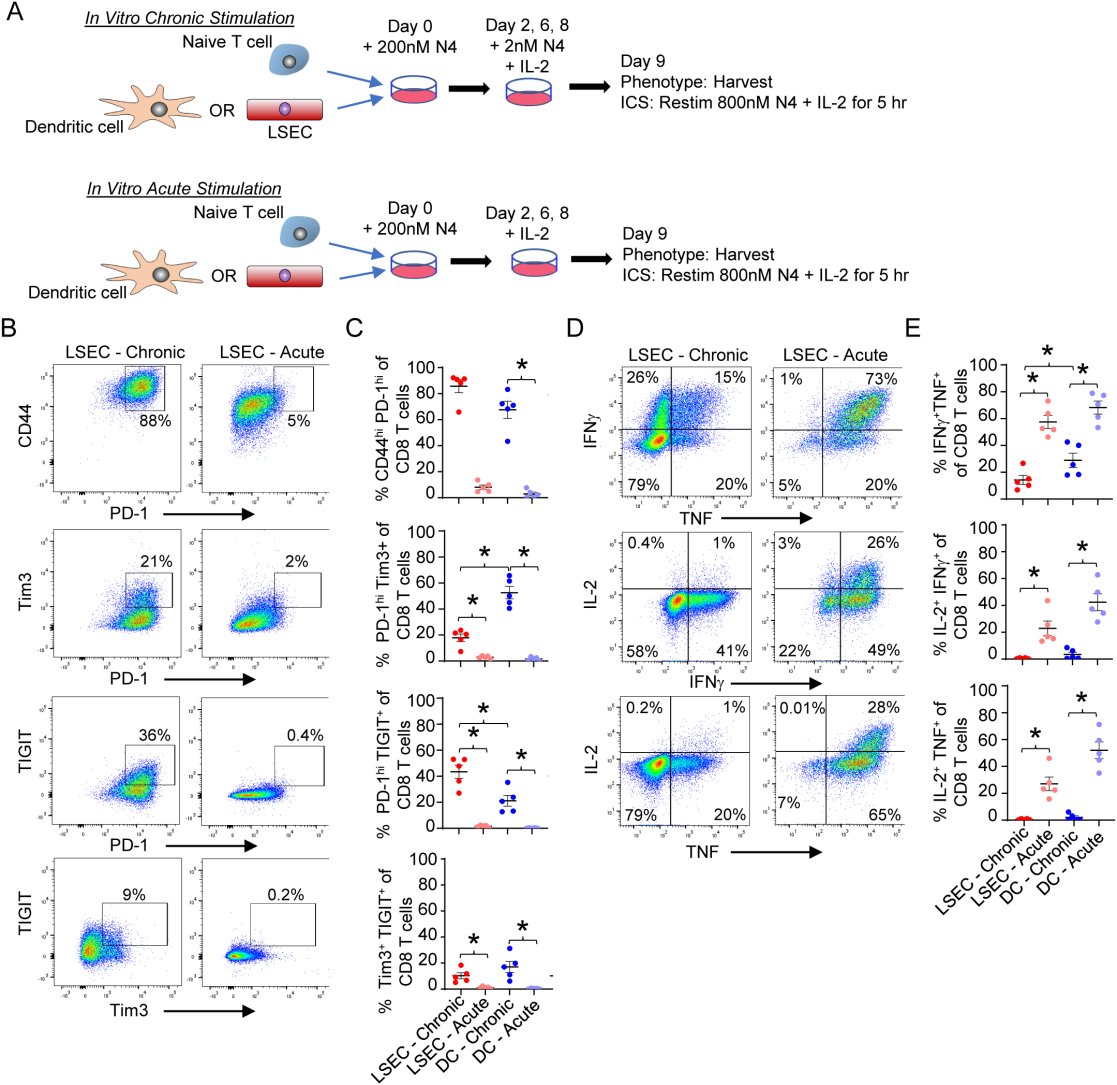
LSEC induce T cell exhaustion in CD8 T cells. **(A)** Schematic of *in vitro* culture for chronic vs acute antigen-specific stimulation of OT-I CD8 T cells. Primary LSEC and splenic DC were separately pulsed with 200nM N4 peptide for 30 min and washed. Naïve OT-I effector CD8 T cells were added to either N4-pulsed LSEC or N4-pulsed DC for 2 days. The cells were then resuspended and replated on day 2, 6, and 8 with IL-2 and either 2nM N4 (chronic stimulation) or no peptide (acute stimulation) and harvested on Day 9. **(B)** Representative phenotypic flow cytometric analysis of OT-I CD8^+^ T cells for PD-1 vs CD44, PD-1 vs Tim3, PD-1 vs TIGIT, and Tim3 vs TIGIT after LSEC chronic (left column) or acute (right) stimulation. **(C)** Summary graphs of each CD8 T cell phenotype after LSEC chronic (red dots) or acute (light red dots) stimulation and DC chronic (blue dots) or acute (light blue dots) stimulation. **(D)** Representative flow cytometric analysis of cytokine expression by OT-I CD8^+^ T cells after LSEC chronic (left column) or acute (right column) stimulation. IFNγ vs TNF (top), IL-2 vs IFNγ (middle) and IL-2 vs TNF (bottom). **(E)** Summary graphs of frequency of cytokine expression by OT-I CD8 T cells after LSEC chronic (red dots) or acute (light red dots) stimulation and DC chronic (blue dots) or acute (light blue dots) stimulation. Summary graphs comparing chronic and acute stimulations of OT-I CD8 T cells by either LSEC or DC are average frequencies, ± SEM. *P ≤ 0.05, Student’s paired t test, n = 5 independent experiments.

The ability of LSEC to induce T cell exhaustion has not been previously explored. However, the increased phenotypic exhaustion induced by DEN/CCl_4_ treatment (**Figure 2**) as well as the corresponding increase of immunoregulatory molecules on LSEC in the same livers (**Figure 3**) suggest that LSEC could induce T cell exhaustion. To directly assess the ability of LSEC to induce T cell exhaustion, we used an established *in vitro* culture system previously used to evaluate DC-mediated induction of T cell exhaustion via a low dose chronic stimulation (48). We first confirmed DC were able to promote T cell exhaustion by low dose stimulation as previously described (48). Naïve OT-I T cells were cultured with isolated splenic DC under conditions where antigen-specific T cells were either chronically or acutely stimulated over 9 days. Both chronic and acute stimulations consisted of an initial treatment of DC with 200 nM N4 peptide prior to culturing with naïve CD8 OT-I T cells for 2 days. On days 2, 6, and 8, fresh IL-2 was added with low dose 2 nM N4 (chronic stimulation) or without N4 (acute stimulation) (**Figure 6A**). On day 9, CD8 T cells activated by DC under chronic stimulation conditions displayed an exhaustion phenotype as indicated by elevated expression of PD-1, TIGIT, and Tim3 and an increased percent of CD44^hi^PD-1^hi^, PD-1^hi^TIGIT^+^, PD-1^hi^Tim3^+^, and TIGIT^+^Tim3^+^ cells of CD8 T cells. In contrast, CD8 T cells acutely stimulated by DC expressed minimal to none of these exhaustion markers (**Figure 6C**). A hallmark of impaired function by exhausted T cells, is the inability of re-stimulated effector CD8 T cells to produce multiple cytokines simultaneously (48). To demonstrate T cell exhaustion functionally, chronically and acutely stimulated CD8 T cells were again restimulated in an antigen-specific manner with N4 and IL-2 on day 9 for 5 hours prior to profiling cytokine expression by flow cytometric intracellular analysis. As expected, DC-mediated chronic stimulation of T cells resulted in a significant decrease in the percent of IFNγ^+^TNF^+^, IL-2^+^IFNγ^+^, and IL-2^+^TNF^+^ CD8 OT-I T cells compared to DC-mediated acute stimulation of T cells, demonstrating that *in vitro* DC chronic stimulation of antigen-specific CD8 effector T cells results in the generation of exhausted T cells (**Figure 6E**). We next determined if LSEC, under similar chronic stimulation conditions, could also induce antigen-specific CD8 T cell exhaustion. Our results demonstrate that naïve OT-I CD8 T cells activated by LSEC presenting N4 peptide and subsequently stimulated in the presence of low dose peptide (chronic stimulation) resulted in phenotypically exhausted CD8 T cells. Specifically, chronic stimulation of OT-I CD8 T cells by LSEC resulted in a significant increase of PD-1^hi^TIGIT^+^, PD1^hi^Tim3^+^, and TIGIT^+^Tim3^+^ CD8 T cell populations compared to acutely stimulated T cells (**Figures 6B, C**). Interestingly, chronic stimulation by LSEC induced a higher percent of CD8 T cells that were PD-1^hi^TIGIT^+^ compared to DC chronic stimulation which instead induced a higher percent of PD-1^hi^Tim3^+^ CD8 T cells, suggesting that LSEC preferentially increase the expression of TIGIT on exhausted T cells compared to Tim3. Nonetheless, OT-I CD8 T cells chronically stimulated by LSEC were also functionally exhausted as demonstrated by their significantly impaired ability to express diverse cytokines after antigen-specific stimulation (**Figures 6D, E**). Here, chronically stimulated OT-I CD8 T cells harbored decreased percentages of IFNγ^+^TNF^+^, IL-2^+^IFNγ^+^, and IL-2^+^TNF^+^ polyfunctional CD8 OT-I T cells compared to LSEC-mediated acute stimulation of T cells (**Figures 6D, E**). Thus, these findings demonstrate that LSEC are able to induce CD8 T cell exhaustion under chronic stimulation conditions with low dose antigen. Together, the results in our study show that LSEC-mediated antigen-specific stimulation significantly impacts T cell functions. Indeed, the DEN/CCl_4_ model demonstrates that, during the early stages of tumor development including liver injury and fibrosis, LSEC expression of immunoregulatory molecules increases, and this increase corresponds with the generation of exhausted hepatic CD8 and CD4 T cells. Furthermore, LSEC alter the function of T cells at various stages of T cell development including altering DC-mediated activation of T cells, inhibiting effector CD8 T cell cytolytic activity while increasing IFNγ production, and by inducing T cell exhaustion under chronic stimulation conditions. Thus, LSEC act to temper T cell activation and function and as such appear as gatekeepers to T cell function that likely impact hepatic immunosurveillance and the development of cancer.

## Discussion

The sheer abundance and strategic location of LSEC within the liver allow LSEC to be key regulators of both hepatic function and immune regulation. The proximity of LSEC to hepatocytes, HSC, and KC enable LSEC to regulate hepatic pathologies including fibrosis, which is initiated by LSEC-hepatocyte interactions that lead to activation of HSC and KC to drive early fibrotic reprogramming (63–65). LSEC also dynamically interact with immune cells, including T cells, as these cells pass through the LSEC-lined sinusoids (19–21). Moreover, the enhanced scavenger ability and capacity to cross-present antigen permit LSEC to internalize and present antigen 100-fold more efficiently than proximal DC (20). Therefore, we propose that liver injury and fibrosis induce LSEC to become more immunosuppressive and thereby contribute to HCC progression. Indeed, we show here that LSEC induce dysfunction of CD8 T cells at various stages of differentiation/activation and that fibrosis results in T cell exhaustion which likely contributes to HCC development.

In this study, we sought to identify the immunophenotypic changes that were directly associated with liver fibrosis. Specifically, we used a model that uncouples the effects of chronic infection, steatosis, and alcohol-induced damage from the establishment of fibrosis (32, 38, 39). We demonstrate that fibrosis (caused by CCl_4_), combined with DNA damage (caused by DEN), does indeed induce a CD8 and CD4 T cell exhausted phenotype indicated here by a significant upregulation of inhibitory receptors associated with T cell exhaustion. DEN-induced DNA damage alone (66) is not sufficient to promote T cell exhaustion in the time-frame assessed here, indicating that liver injury alone is not sufficient to promote exhaustion, and that malignant cells can be effectively controlled by the immune system in this scenario. However, upon induction of fibrosis by CCl_4_ in the DEN/CCl_4_ model, T cell exhaustion correlated with an increased expression of immunosuppressive molecules by LSEC including PD-L1, ICAM-1, and MHC-I. Increased PD-L1 expression by various cell types, including tumor cells, has been extensively shown to reduce T cell function and promote exhaustion (1, 16, 67, 68). Upregulation of ICAM-1 expression on hepatic stellate cells has also previously been shown to impair T cell function by introducing competing immune synapses (69). Increased expression of both ICAM1 and MHC-I by LSEC retains CD8 T cells in the liver (21, 51–53). Thus, the combined increased expression of PD-L1, ICAM-1, and MHC-I likely increases interactions between LSEC and CD8 T cells and retains CD8 T cells in the liver leading to T cell exhaustion. The source of low dose antigen required to induce T cell exhaustion in this model is likely from DEN-induced DNA-damaged cells and could be envisioned to be alpha fetoprotein or glypican-3 (70–72). The most abundant antigen presenting cells in the liver include LSEC, KC, and IM (18), and we show here that LSEC, but not KC or IM, increase expression of immunosuppressive molecules. Further studies will be needed to determine if KC or IM contribute to T cell exhaustion by mechanisms not assessed here. Nonetheless, these results suggest that fibrosis enhances the immunosuppressive capabilities of LSEC resulting in T cell exhaustion.

The antigen-specific stimulation of naïve CD8 T cells by antigen presenting cells leads to their differentiation into effector cytotoxic CD8 T cells that, upon encounter with a target cell presenting the same specific antigen, results in directed target cell killing. We demonstrate here that LSEC presenting cognate antigen, to previously activated effector CD8 T cells that recognize the same antigen, severely impeded the killing of target cells by these cytotoxic effector T cells. The scavenging ability of LSEC prevents the release of dietary antigens into the periphery as well as the unwanted uptake of dietary antigens by hepatic DC, both of which could lead to adverse immune responses (19, 20, 23, 53, 64). Nonetheless, the presentation of cognate antigen by LSEC to effector cells, and the resulting inhibition of killing ability, likely represents a self-preservation mechanism to prevent effector cell-mediated LSEC death and liver injury under physiologically normal conditions (62). However, this self-preservation mechanism will likely impact the killing of malignant hepatocytes by effectors in an environment where the hepatocytes are releasing tumor antigens that are being taken up and presented by LSEC to effector cells that had been previously activated in the lymph nodes. Importantly, presentation of an irrelevant peptide by LSEC to effector CD8 T cells did not impede the killing of antigen-specific target cells by these cytotoxic cells. Moreover, despite preventing target cell killing, LSEC presenting cognate antigen increased effector cell IFNγ production by 50-100 fold during the 6 hour coculture. Target cell killing requires the formation of a functional immune synapse between target cells and effectors that is driven by peptide-pulsed MHC-I interactions with cognate TCR, respectively (47). The results presented here suggest that LSEC outcompete with target cells for interaction with the TCR expressed on effector cells, thereby inhibiting killing of the target cells and enhancing IFNγ production. The mechanism driving the substantial increase in IFNγ production by effector cells is unclear, however, IFNγ production has been shown to increase PD-L1 expression (73), thereby further contributing to an immunosuppressive environment. Therefore, LSEC, as protectors and repairers of liver injury, likely contribute to the development of HCC by impairing the selective killing of damaged/malignant hepatocytes by effector CD8 T cells.

LSEC are within close proximity to DC within the portal regions of the liver (59, 60), and we demonstrate here that simultaneous presentation of the same antigen by LSEC and DC results in a unique activation and functional phenotype of naïve T cells compared to naïve T cells activated by either LSEC or DC alone. A previous study, aimed at identifying antigen presentation-independent effects of LSEC on DC-mediated naïve T cell activation, demonstrated that LSEC with mismatched MHC, that were unable to stimulate T cells via the antigen receptor, were nevertheless able to impair DC-mediated naïve T cell proliferation, activation, and function (26). Here, we sought to assess naive T cell activation when LSEC and DC are both presenting the same cognate antigen, a possible scenario given their close proximity within the portal regions of the liver. Although proliferation of naïve T cells upon activation by DC and LSEC together did not change after stimulation by either cell type alone, naïve T cells activated by LSEC and DC together failed to upregulate CD44 and PD-1 as efficiently as T cells activated by DC alone, however CD25 was upregulated to a similar level as DC alone. Since the naïve T cells activated by both LSEC and DC remained as a single population, it is likely that the naïve T cells are interacting with both LSEC and DC with the TCR competing for MHC I and other costimulatory molecules on both the LSEC and DC. It is also possible that LSEC and DC have direct effects on each other that alters their ability to fully activate naïve T cells. Importantly, IL-2 and TNF production by naïve T cells activated by LSEC and DC together, displayed an intermediate phenotype between LSEC or DC alone, while IFNγ production remained unchanged. Together these results suggest that the ability of LSEC to present the same antigen as nearby DC to cognate naïve T cells results in an altered phenotype and function of the resulting activated T cells. These dysfunctional T cells are unlikely to provide efficient immunosurveillance and therefore may contribute to HCC development.

The ability of LSEC to induce functional T cell exhaustion, suggested by the DEN/CCl_4_ model (**Figures 2**, **3**), was confirmed using an *in vitro* chronic stimulation assay (**Figure 6**). Utilizing a previously described *in vitro* method to induce T cell exhaustion with chronic low dose antigen presentation via DC (48), we show that LSEC not only induce a T cell exhaustion phenotype (high PD-1, Tim3, and TIGIT expression), but LSEC also decrease the percent of dual-cytokine producing T cells (i.e. polyfunctional) upon restimulation, a feature of functional T cell exhaustion (48). Interestingly, comparing DC- vs LSEC-mediated T cell exhaustion, we found that LSEC induce an increased percentage of TIGIT+ T cells compared to DC, and conversely DC induce an increased percentage of Tim3+ T cells compared to LSEC. Recent findings suggest that TIGIT expression occurs on PD-1^hi^, but not PD-1^lo^, CD8 T cells, and that blocking the TIGIT signaling pathway can reverse T cell exhaustion (15–17, 49). Accordingly, immunotherapy targeting both PD-1 and TIGIT has shown increased efficacy in reversing T cell exhaustion compared to either therapy alone (15–17, 49). Therefore, the understanding that LSEC do indeed promote T cell exhaustion at the fibrotic stage of HCC, and that many of these exhausted T cells express TIGIT, could lead to further investigations into the use of anti-TIGIT antibodies as an effective co-immunotherapy during advanced stages of fibrosis and cirrhosis to prevent or delay the progression of HCC. The induction of T cell exhaustion does not result in complete inhibition of T cell function, but rather an impaired T cell function, which is thought to prevent immunopathology that could harm otherwise healthy tissues while attempting to limit, but not eliminate, infectious or malignant progression (62). Thus, LSEC may induce T cell exhaustion to limit LSEC-mediated cell death and liver injury, similar to LSEC-mediated inhibition of effector T cell function.

Together, the results in this study demonstrate that LSEC alter CD8 T cell function at various stages of T cell differentiation by modifying the level of T cell activation initiated by other antigen presenting cells, the ability of effector CD8 T cells to kill target cells, and the types and amounts of cytokines produced by T cells upon initial activation or restimulation. Importantly, T cell exhaustion is associated with liver disease prior to HCC formation, mediated in part by LSEC, indicating the potential of targeted interventions, such as co-immunotherapy with PD-1 and TIGIT, for those affected by chronic liver disease.

## Data Availability

The datasets presented in this article are readily available. Requests to access these datasets should be directed to kimberly.kremer@cuanschutz.edu.

## Supplementary material

Supplementary Figure 1 DEN/CCl_4_ treatment increases the percent of CD4^+^FoxP3^+^T cells present in the liver. **(A)** Representative flow cytometric analysis of NPC from DEN/olive oil- (left) or DEN/CCl_4_-treated (right) showing percentage of potential Tregs (CD4^+^FoxP3^+^) among CD4^+^ T cells. **(B)** Summary graphs of CD4^+^FoxP3^+^ Tregs was determined between DEN/olive oil- (red) and DEN/CCl_4_-treated (blue) mice, ± SEM. *P ≤ 0.05, Student’s unpaired t test, n = 2 independent experiments, with 5-8 mice per experiment.

Supplementary Figure 2 DEN/CCl_4_ treatment does not increase inhibitory receptors on KC or IM. NPC from DEN/olive oil- (red) or DEN/CCl_4_-treated (blue) mice were evaluated by flow cytometry for the expression of immunosuppressive molecules by KC (CD45^+^CD14^+^CD11b^lo^F4/80^hi^ of NPC) and IM (CD45^+^CD14^+^CD11b^hi^F4/80^lo^ of NPC). **(A)** Representative plots, MFI normalized to the average of the olive oil control, are shown for PD-L1, ICAM-1, FasL, H2-K^b^, I-A^b^ and CD80 expression on KC. **(B, C)** Summary graphs of the expression of indicated surface receptors on **(B)** KC and **(C)** IM after DEN/olive oil (red) or DEN/CCl_4_ (blue) treatment, shown normalized to the average of DEN/olive oil-treated mice, ± SEM. *P ≤ 0.05, Student’s unpaired t test, n = 2 independent experiments, with 5-8 mice per experiment.

## References

[1] Ma J Zheng B Goswami S Meng L Zhang D Cao C. PD1(Hi) CD8(+) T cells correlate with exhausted signature and poor clinical outcome in hepatocellular carcinoma. J Immunother Cancer. (2019) 7:331. doi: 10.1186/s40425-019-0814-7 31783783 PMC6884778

[2] Zhu Y Tan H Wang J Zhuang H Zhao H Lu X. Molecular insight into T cell exhaustion in hepatocellular carcinoma. Pharmacol Res. (2024) 203:107161. doi: 10.1016/j.phrs.2024.107161 38554789

[3] Barsch M Salie H Schlaak AE Zhang Z Hess M Mayer LS. T-cell exhaustion and residency dynamics inform clinical outcomes in hepatocellular carcinoma. J Hepatol. (2022) 77:397–409. doi: 10.1016/j.jhep.2022.02.032 35367533

[4] Zheng C Zheng L Yoo JK Guo H Zhang Y Guo X. Landscape of infiltrating T cells in liver cancer revealed by single-cell sequencing. Cell. (2017) 169:1342–1356.e16. doi: 10.1016/j.cell.2017.05.035 28622514

[5] Ahmadzadeh M Johnson LA Heemskerk B Wunderlich JR Dudley ME White DE. Tumor antigen-specific CD8 T cells infiltrating the tumor express high levels of PD-1 and are functionally impaired. Blood. (2009) 114:1537–44. doi: 10.1182/blood-2008-12-195792 PMC2927090 19423728

[6] Dhar D Baglieri J Kisseleva T Brenner DA. Mechanisms of liver fibrosis and its role in liver cancer. Exp Biol Med (Maywood). (2020) 245:96–108. doi: 10.1177/1535370219898141 31924111 PMC7016420

[7] Li D Sedano S Allen R Gong J Cho M Sharma S. Current treatment landscape for advanced hepatocellular carcinoma: patient outcomes and the impact on quality of life. Cancers (Basel). (2019) 11. doi: 10.3390/cancers11060841 PMC6627588 31216701

[8] Raskov H Orhan A Christensen JP Gogenur I. Cytotoxic CD8(+) T cells in cancer and cancer immunotherapy. Br J Cancer. (2021) 124:359–67. doi: 10.1038/s41416-020-01048-4 PMC7853123 32929195

[9] Zhang N Bevan MJ. CD8(+) T cells: foot soldiers of the immune system. Immunity. (2011) 35:161–8. doi: 10.1016/j.immuni.2011.07.010 PMC3303224 21867926

[10] Zhang CY Liu S Yang M. Regulatory T cells and their associated factors in hepatocellular carcinoma development and therapy. World J Gastroenterol. (2022) 28:3346–58. doi: 10.3748/wjg.v28.i27.3346 PMC9346458 36158267

[11] Dolina JS Van Braeckel-Budimir N Thomas GD Salek-Ardakani S. CD8(+) T cell exhaustion in cancer. Front Immunol. (2021) 12:715234. doi: 10.3389/fimmu.2021.715234 34354714 PMC8330547

[12] Hao L Li S Hu X. New insights into T-cell exhaustion in liver cancer: from mechanism to therapy. J Cancer Res Clin Oncol. (2023) 149:12543–60. doi: 10.1007/s00432-023-05083-5 PMC11797504 37423958

[13] Hackstein CP Spitzer J Symeonidis K Horvatic H Bedke T Steglich B. Interferon-induced IL-10 drives systemic T-cell dysfunction during chronic liver injury. J Hepatol. (2023) 79:150–66. doi: 10.1016/j.jhep.2023.02.026 36870611

[14] Brown ZJ Heinrich B Greten TF. Mouse models of hepatocellular carcinoma: an overview and highlights for immunotherapy research. Nat Rev Gastroenterol Hepatol. (2018) 15:536–54. doi: 10.1038/s41575-018-0033-6 29904153

[15] Ge Z Peppelenbosch MP Sprengers D Kwekkeboom J. TIGIT, the next step towards successful combination immune checkpoint therapy in cancer. Front Immunol. (2021) 12:699895. doi: 10.3389/fimmu.2021.699895 34367161 PMC8339559

[16] Ge Z Zhou G Campos Carrascosa L Gausvik E Boor PPC Noordam L. TIGIT and PD1 Co-blockade Restores ex vivo Functions of Human Tumor-Infiltrating CD8(+) T Cells in Hepatocellular Carcinoma. Cell Mol Gastroenterol Hepatol. (2021) 12:443–64. doi: 10.1016/j.jcmgh.2021.03.003 PMC8255944 33781741

[17] Wei YY Fan J Shan MX Yin DD Wang LL Ye W. TIGIT marks exhausted T cells and serves as a target for immune restoration in patients with chronic HBV infection. Am J Transl Res. (2022) 14:942–54. PMC8902551 35273697

[18] Horst AK Neumann K Diehl L Tiegs G. Modulation of liver tolerance by conventional and nonconventional antigen-presenting cells and regulatory immune cells. Cell Mol Immunol. (2016) 13:277–92. doi: 10.1038/cmi.2015.112 PMC4856800 27041638

[19] Wilkinson AL Qurashi M Shetty S. The role of sinusoidal endothelial cells in the axis of inflammation and cancer within the liver. Front Physiol. (2020) 11:990. doi: 10.3389/fphys.2020.00990 32982772 PMC7485256

[20] Knolle PA Wohlleber D. Immunological functions of liver sinusoidal endothelial cells. Cell Mol Immunol. (2016) 13:347–53. doi: 10.1038/cmi.2016.5 PMC4856811 27041636

[21] Warren A Le Couteur DG Fraser R Bowen DG McCaughan GW Bertolino P. T lymphocytes interact with hepatocytes through fenestrations in murine liver sinusoidal endothelial cells. Hepatology. (2006) 44:1182–90. doi: 10.1002/hep.21378 17058232

[22] Ye B Liu X Li X Kong H Tian L Chen Y. T-cell exhaustion in chronic hepatitis B infection: current knowledge and clinical significance. Cell Death Dis. (2015) 6:e1694. doi: 10.1038/cddis.2015.42 25789969 PMC4385920

[23] Limmer A Ohl J Kurts C Ljunggren HG Reiss Y Groettrup M. Efficient presentation of exogenous antigen by liver endothelial cells to CD8+ T cells results in antigen-specific T-cell tolerance. Nat Med. (2000) 6:1348–54. doi: 10.1038/82161 11100119

[24] Diehl L Schurich A Grochtmann R Hegenbarth S Chen L Knolle PA. Tolerogenic maturation of liver sinusoidal endothelial cells promotes B7-homolog 1-dependent CD8+ T cell tolerance. Hepatology. (2008) 47:296–305. doi: 10.1002/hep.21965 17975811

[25] Schurich A Berg M Stabenow D Bottcher J Kern M Schild HJ. Dynamic regulation of CD8 T cell tolerance induction by liver sinusoidal endothelial cells. J Immunol. (2010) 184:4107–14. doi: 10.4049/jimmunol.0902580 20212092

[26] Schildberg FA Hegenbarth SI Schumak B Scholz K Limmer A Knolle PA. Liver sinusoidal endothelial cells veto CD8 T cell activation by antigen-presenting dendritic cells. Eur J Immunol. (2008) 38:957–67. doi: 10.1002/eji.200738060 18383043

[27] Hochst B Schildberg FA Bottcher J Metzger C Huss S Turler A. Liver sinusoidal endothelial cells contribute to CD8 T cell tolerance toward circulating carcinoembryonic antigen in mice. Hepatology. (2012) 56:1924–33. doi: 10.1002/hep.25844 22610745

[28] Fujita T Narumiya S. Roles of hepatic stellate cells in liver inflammation: a new perspective. Inflammation Regener. (2016) 36:1. doi: 10.1186/s41232-016-0005-6 PMC5721720 29259674

[29] Thompson AI Conroy KP Henderson NC. Hepatic stellate cells: central modulators of hepatic carcinogenesis. BMC Gastroenterol. (2015) 15:63. doi: 10.1186/s12876-015-0291-5 26013123 PMC4445994

[30] Guidotti LG Inverso D Sironi L Di Lucia P Fioravanti J Ganzer L. Immunosurveillance of the liver by intravascular effector CD8(+) T cells. Cell. (2015) 161:486–500. doi: 10.1016/j.cell.2015.03.005 25892224 PMC11630812

[31] Liu S Huang F Ru G Wang Y Zhang B Chen X. Mouse models of hepatocellular carcinoma: classification, advancement, and application. Front Oncol. (2022) 12:902820. doi: 10.3389/fonc.2022.902820 35847898 PMC9279915

[32] Romualdo GR Prata GB da Silva TC Fernandes AAH Moreno FS Cogliati B. Fibrosis-associated hepatocarcinogenesis revisited: Establishing standard medium-term chemically-induced male and female models. PLoS One. (2018) 13:e0203879. doi: 10.1371/journal.pone.0203879 30212575 PMC6136798

[33] Brol MJ Rosch F Schierwagen R Magdaleno F Uschner FE Manekeller S. Combination of CCl(4) with alcoholic and metabolic injuries mimics human liver fibrosis. Am J Physiol Gastrointest Liver Physiol. (2019) 317:G182–94. doi: 10.1152/ajpgi.00361.2018 31188634

[34] Brandon-Warner E Walling TL Schrum LW McKillop IH. Chronic ethanol feeding accelerates hepatocellular carcinoma progression in a sex-dependent manner in a mouse model of hepatocarcinogenesis. Alcohol Clin Exp Res. (2012) 36:641–53. doi: 10.1111/j.1530-0277.2011.01660.x PMC3288433 22017344

[35] Jepsen P Vilstrup H Tarone RE Friis S Sorensen HT. Incidence rates of hepatocellular carcinoma in the U.S. and Denmark: recent trends. Int J Cancer. (2007) 121:1624–6. doi: 10.1002/ijc.v121:7 17557292

[36] Lee GH Nomura K Kitagawa T. Comparative study of diethylnitrosamine-initiated two-stage hepatocarcinogenesis in C3H, C57BL and BALB mice promoted by various hepatopromoters. Carcinogenesis. (1989) 10:2227–30. doi: 10.1093/carcin/10.12.2227 2591012

[37] Tolba R Kraus T Liedtke C Schwarz M Weiskirchen R. Diethylnitrosamine (DEN)-induced carcinogenic liver injury in mice. Lab Anim. (2015) 49:59–69. doi: 10.1177/0023677215570086 25835739

[38] Uehara T Pogribny IP Rusyn I. The DEN and CCl4 -induced mouse model of fibrosis and inflammation-associated hepatocellular carcinoma. Curr Protoc Pharmacol. (2014) 66:14 30 1–14 30 10. doi: 10.1002/0471141755.ph1430s66 PMC4214366 25181010

[39] Uehara T Pogribny IP Rusyn I. The DEN and CCl(4) -induced mouse model of fibrosis and inflammation-associated hepatocellular carcinoma. Curr Protoc. (2021) 1:e211. doi: 10.1002/cpz1.211 34370903 PMC8744072

[40] Weghorst CM Pereira MA Klaunig JE. Strain differences in hepatic tumor promotion by phenobarbital in diethylnitrosamine- and dimethylnitrosamine-initiated infant male mice. Carcinogenesis. (1989) 10:1409–12. doi: 10.1093/carcin/10.8.1409 2752514

[41] Lanaspa MA Andres-Hernando A Orlicky DJ Cicerchi C Jang C Li N. Ketohexokinase C blockade ameliorates fructose-induced metabolic dysfunction in fructose-sensitive mice. J Clin Invest. (2018) 128:2226–38. doi: 10.1172/JCI94427 PMC5983342 29533924

[42] Monks J Orlicky DJ Stefanski AL Libby AE Bales ES Rudolph MC. Maternal obesity during lactation may protect offspring from high fat diet-induced metabolic dysfunction. Nutr Diabetes. (2018) 8:18. doi: 10.1038/s41387-018-0027-z 29695710 PMC5916951

[43] Kleiner DE Brunt EM Van Natta M Behling C Contos MJ Cummings OW. Design and validation of a histological scoring system for nonalcoholic fatty liver disease. Hepatology. (2005) 41:1313–21. doi: 10.1002/hep.20701 15915461

[44] McLoughlin MR Orlicky DJ Prigge JR Krishna P Talago EA Cavigli IR. TrxR1, Gsr, and oxidative stress determine hepatocellular carcinoma Malignancy. Proc Natl Acad Sci U.S.A. (2019) 116:11408–17. doi: 10.1073/pnas.1903244116 PMC6561278 31097586

[45] Karpova Y Orlicky DJ Schmidt EE Tulin AV. Disrupting poly(ADP-ribosyl)ating pathway creates premalignant conditions in mammalian liver. Int J Mol Sci. (2023) 24. doi: 10.3390/ijms242417205 PMC10743425 38139034

[46] Finlon JM Burchill MA Tamburini BAJ. Digestion of the murine liver for a flow cytometric analysis of lymphatic endothelial cells. J Vis Exp. (2019) 143. doi: 10.3791/58621-v PMC6350926 30663671

[47] Kremer KN Buser A Thumkeo D Narumiya S Jacobelli J Pelanda R. LPA suppresses T cell function by altering the cytoskeleton and disrupting immune synapse formation. Proc Natl Acad Sci U.S.A. (2022) 119:e2118816119. doi: 10.1073/pnas.2118816119 35394866 PMC9169816

[48] Wu JE Manne S Ngiow SF Baxter AE Huang H Freilich E. *In vitro* modeling of CD8(+) T cell exhaustion enables CRISPR screening to reveal a role for BHLHE40. Sci Immunol. (2023) 8:eade3369. doi: 10.1126/sciimmunol.ade3369 37595022 PMC11975459

[49] Ostroumov D Duong S Wingerath J Woller N Manns MP Timrott K. Transcriptome profiling identifies TIGIT as a marker of T-cell exhaustion in liver cancer. Hepatology. (2021) 73:1399–418. doi: 10.1002/hep.31466 32716559

[50] Kaczmarek J Homsi Y van Uum J Metzger C Knolle PA Kolanus W. Liver sinusoidal endothelial cell-mediated CD8 T cell priming depends on co-inhibitory signal integration over time. PLoS One. (2014) 9:e99574. doi: 10.1371/journal.pone.0099574 24924593 PMC4055751

[51] McNamara HA Cai Y Wagle MV Sontani Y Roots CM Miosge LA. Up-regulation of LFA-1 allows liver-resident memory T cells to patrol and remain in the hepatic sinusoids. Sci Immunol. (2017) 2. doi: 10.1126/sciimmunol.aaj1996 PMC5505664 28707003

[52] Mehal WZ Juedes AE Crispe IN. Selective retention of activated CD8+ T cells by the normal liver. J Immunol. (1999) 163:3202–10. doi: 10.4049/jimmunol.163.6.3202 10477588

[53] von Oppen N Schurich A Hegenbarth S Stabenow D Tolba R Weiskirchen R. Systemic antigen cross-presented by liver sinusoidal endothelial cells induces liver-specific CD8 T-cell retention and tolerization. Hepatology. (2009) 49:1664–72. doi: 10.1002/hep.22795 19205034

[54] Wu K Kryczek I Chen L Zou W Welling TH. Kupffer cell suppression of CD8+ T cells in human hepatocellular carcinoma is mediated by B7-H1/programmed death-1 interactions. Cancer Res. (2009) 69:8067–75. doi: 10.1158/0008-5472.CAN-09-0901 PMC4397483 19826049

[55] You Q Cheng L Kedl RM Ju C. Mechanism of T cell tolerance induction by murine hepatic Kupffer cells. Hepatology. (2008) 48:978–90. doi: 10.1002/hep.22395 PMC2600585 18712788

[56] Knolle PA Germann T Treichel U Uhrig A Schmitt E Hegenbarth S. Endotoxin down-regulates T cell activation by antigen-presenting liver sinusoidal endothelial cells. J Immunol. (1999) 162:1401–7. doi: 10.4049/jimmunol.162.3.1401 9973395

[57] Knolle PA Uhrig A Hegenbarth S Loser E Schmitt E Gerken G. IL-10 down-regulates T cell activation by antigen-presenting liver sinusoidal endothelial cells through decreased antigen uptake via the mannose receptor and lowered surface expression of accessory molecules. Clin Exp Immunol. (1998) 114:427–33. doi: 10.1046/j.1365-2249.1998.00713.x PMC1905120 9844054

[58] Ramachandran P Pellicoro A Vernon MA Boulter L Aucott RL Ali A. Differential Ly-6C expression identifies the recruited macrophage phenotype, which orchestrates the regression of murine liver fibrosis. Proc Natl Acad Sci U.S.A. (2012) 109:E3186–95. doi: 10.1073/pnas.1119964109 PMC3503234 23100531

[59] Lau AH Thomson AW. Dendritic cells and immune regulation in the liver. Gut. (2003) 52:307–14. doi: 10.1136/gut.52.2.307 PMC1774973 12524419

[60] Mendez-Sanchez N Cordova-Gallardo J Barranco-Fragoso B Eslam M. Hepatic dendritic cells in the development and progression of metabolic steatohepatitis. Front Immunol. (2021) 12:641240. doi: 10.3389/fimmu.2021.641240 33833761 PMC8021782

[61] Hogquist KA Jameson SC Heath WR Howard JL Bevan MJ Carbone FR. T cell receptor antagonist peptides induce positive selection. Cell. (1994) 76:17–27. doi: 10.1016/0092-8674(94)90169-4 8287475

[62] Mueller SN Matloubian M Clemens DM Sharpe AH Freeman GJ Gangappa S. Viral targeting of fibroblastic reticular cells contributes to immunosuppression and persistence during chronic infection. Proc Natl Acad Sci U.S.A. (2007) 104:15430–5. doi: 10.1073/pnas.0702579104 PMC2000533 17878315

[63] Alirzayeva E Neumann G Horst W Allahverdiyeva Y Specht A Alizade V. Multiple mechanisms of heavy metal tolerance are differentially expressed in ecotypes of Artemisia fragrans. Environ pollut. (2017) 220:1024–35. doi: 10.1016/j.envpol.2016.11.041 27890587

[64] Du W Wang L. The crosstalk between liver sinusoidal endothelial cells and hepatic microenvironment in NASH related liver fibrosis. Front Immunol. (2022) 13:936196. doi: 10.3389/fimmu.2022.936196 35837401 PMC9274003

[65] Kostallari E Shah VH. Angiocrine signaling in the hepatic sinusoids in health and disease. Am J Physiol Gastrointest Liver Physiol. (2016) 311:G246–51. doi: 10.1152/ajpgi.00118.2016 PMC5007289 27288423

[66] Schneider C Teufel A Yevsa T Staib F Hohmeyer A Walenda G. Adaptive immunity suppresses formation and progression of diethylnitrosamine-induced liver cancer. Gut. (2012) 61:1733–43. doi: 10.1136/gutjnl-2011-301116 PMC4533880 22267597

[67] Lebosse F Gudd C Tunc E Singanayagam A Nathwani R Triantafyllou E. CD8(+)T cells from patients with cirrhosis display a phenotype that may contribute to cirrhosis-associated immune dysfunction. EBioMedicine. (2019) 49:258–68. doi: 10.1016/j.ebiom.2019.10.011 PMC6945243 31678004

[68] Pfister D Nunez NG Pinyol R Govaere O Pinter M Szydlowska M. NASH limits anti-tumour surveillance in immunotherapy-treated HCC. Nature. (2021) 592:450–6. doi: 10.1038/s41586-021-03362-0 PMC8046670 33762733

[69] Schildberg FA Wojtalla A Siegmund SV Endl E Diehl L Abdullah Z. Murine hepatic stellate cells veto CD8 T cell activation by a CD54-dependent mechanism. Hepatology. (2011) 54:262–72. doi: 10.1002/hep.24352 21488077

[70] Qiu B Wang J Yu Y Zhen C Gu J Liu W. DJ-1 promotes development of DEN-induced hepatocellular carcinoma and proliferation of liver cancer cells. Oncotarget. (2017) 8:8499–511. doi: 10.18632/oncotarget.14293 PMC5352417 28036277

[71] Yi X Long L Yang C Lu Y Cheng M. Maotai ameliorates diethylnitrosamine-initiated hepatocellular carcinoma formation in mice. PLoS One. (2014) 9:e93599. doi: 10.1371/journal.pone.0093599 24690765 PMC3972115

[72] Chen T Dai X Dai J Ding C Zhang Z Lin Z. AFP promotes HCC progression by suppressing the HuR-mediated Fas/FADD apoptotic pathway. Cell Death Dis. (2020) 11:822. doi: 10.1038/s41419-020-03030-7 33009373 PMC7532541

[73] Chen S Crabill GA Pritchard TS McMiller TL Wei P Pardoll DM. Mechanisms regulating PD-L1 expression on tumor and immune cells. J Immunother Cancer. (2019) 7:305. doi: 10.1186/s40425-019-0770-20 31730010 PMC6858680

