# Supplementary figures and images for: Liver sinusoidal endothelial cells regulate the balance between hepatic immunosuppression and immunosurveillance
Source: Front Immunol. 2025 Jan 17;15:1497788. doi: 10.3389/fimmu.2024.1497788 (PMC11782242; doi:10.3389/fimmu.2024.1497788)

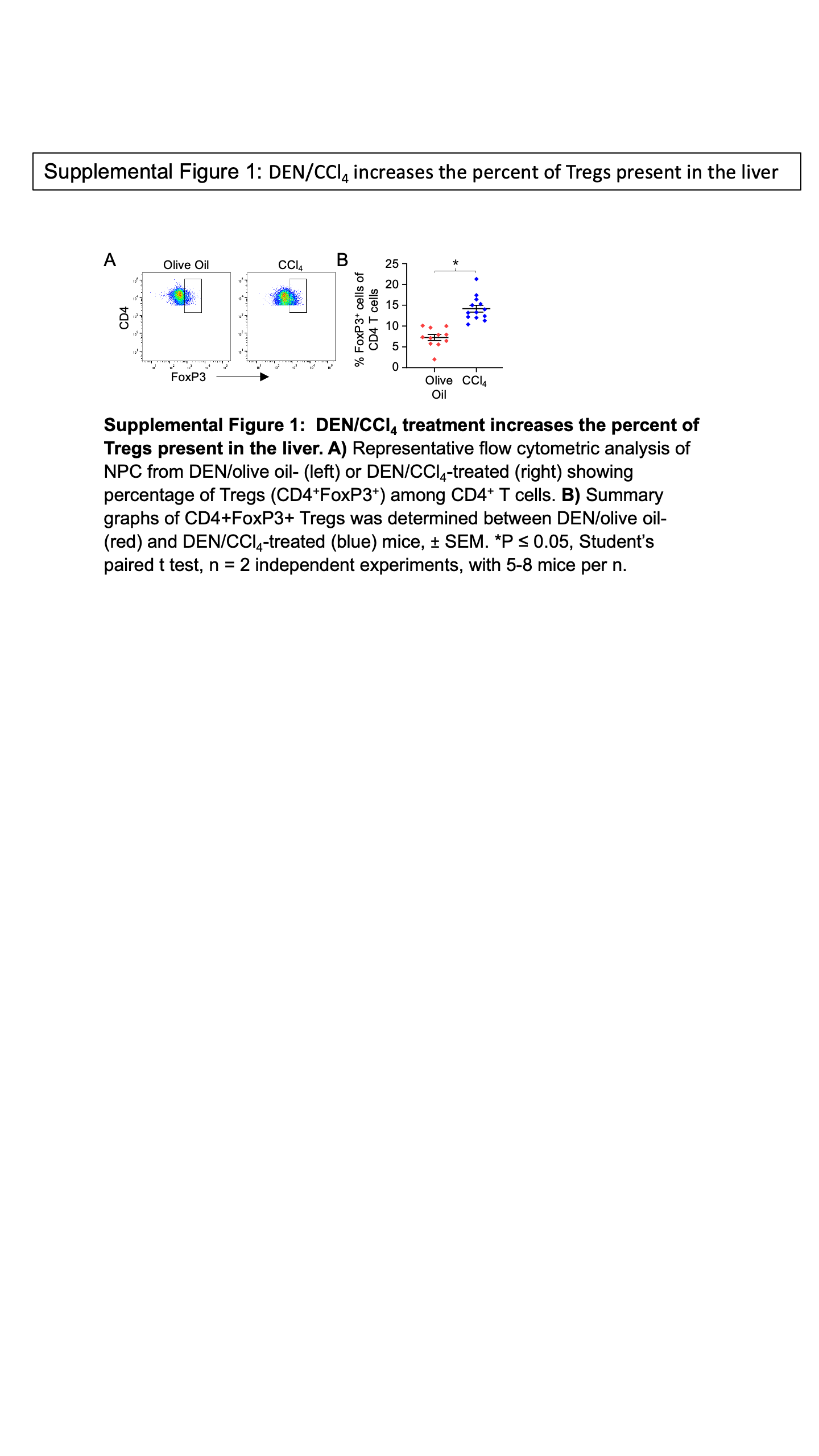

Supplement: Supplementary Figure 1 — DEN/CCl4 treatment increases the percent of CD4+FoxP3+T cells present in the liver. (A) Representative flow cytometric analysis of NPC from DEN/olive oil- (left) or DEN/CCl4-treated (right) showing percentage of potential Tregs (CD4+FoxP3+) among CD4+ T cells. (B) Summary graphs of CD4+FoxP3+ Tregs was determined between DEN/olive oil- (red) and DEN/CCl4-treated (blue) mice, ± SEM. *P ≤ 0.05, Student’s unpaired t test, n = 2 independent experiments, with 5-8 mice per experiment. [file Image1.tiff]

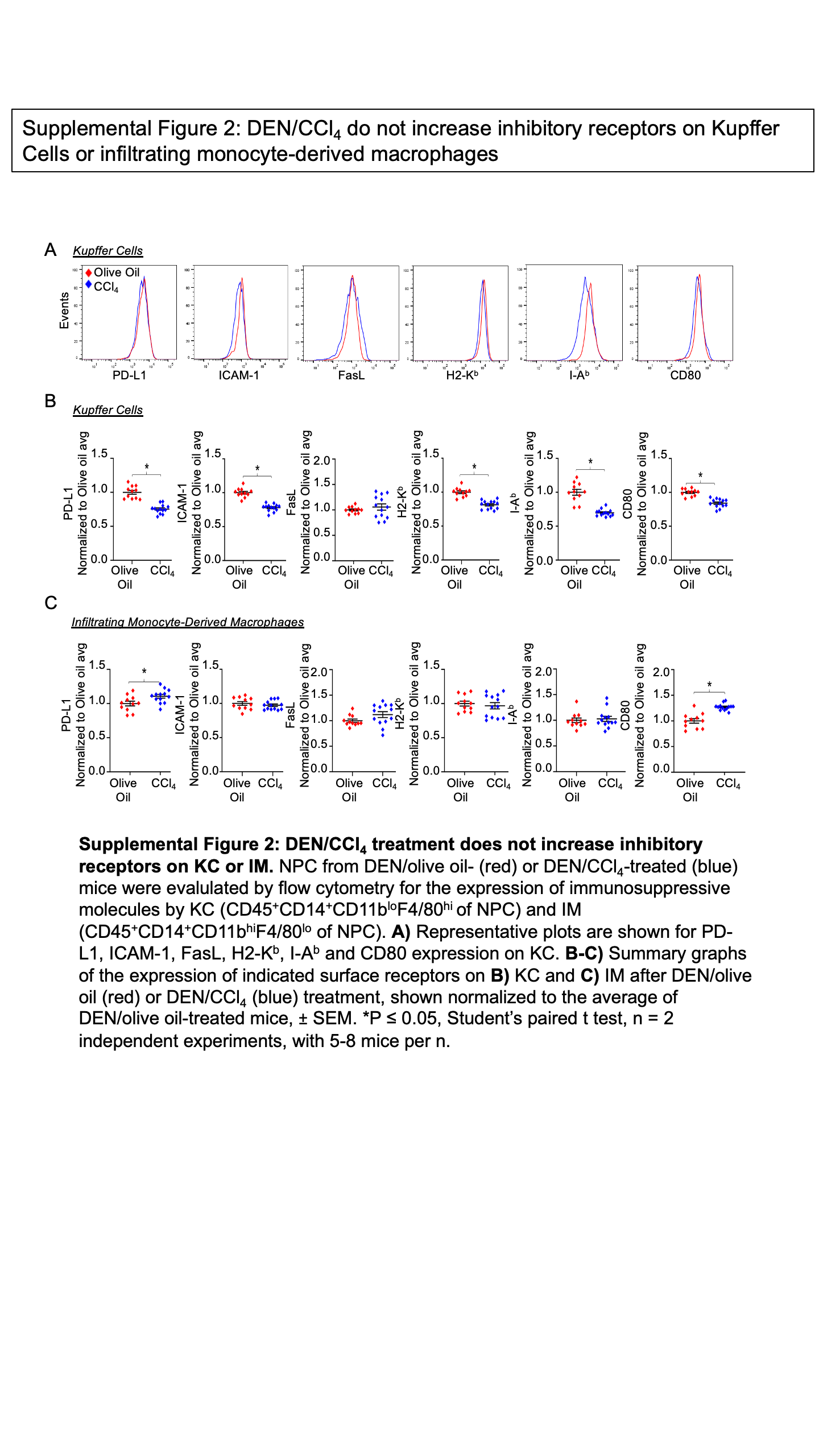

Supplement: Supplementary Figure 2 — DEN/CCl4 treatment does not increase inhibitory receptors on KC or IM. NPC from DEN/olive oil- (red) or DEN/CCl4-treated (blue) mice were evaluated by flow cytometry for the expression of immunosuppressive molecules by KC (CD45+CD14+CD11bloF4/80hi of NPC) and IM (CD45+CD14+CD11bhiF4/80lo of NPC). (A) Representative plots, MFI normalized to the average of the olive oil control, are shown for PD-L1, ICAM-1, FasL, H2-Kb, I-Ab and CD80 expression on KC. (B, C) Summary graphs of the expression of indicated surface receptors on (B) KC and (C) IM after DEN/olive oil (red) or DEN/CCl4 (blue) treatment, shown normalized to the average of DEN/olive oil-treated mice, ± SEM. *P ≤ 0.05, Student’s unpaired t test, n = 2 independent experiments, with 5-8 mice per experiment. [file Image2.tiff]
